# Functional annotation of human cytomegalovirus gene products: an update

**DOI:** 10.3389/fmicb.2014.00218

**Published:** 2014-05-19

**Authors:** Ellen Van Damme, Marnix Van Loock

**Affiliations:** Janssen Infectious Diseases BVBA, Therapeutic Area of Infectious DiseasesBeerse, Belgium

**Keywords:** human cytomegalovirus, function, annotation, genome, gene expression

## Abstract

Human cytomegalovirus is an opportunistic double-stranded DNA virus with one of the largest viral genomes known. The 235 kB genome is divided in a unique long (UL) and a unique short (US) region which are flanked by terminal and internal repeats. The expression of HCMV genes is highly complex and involves the production of protein coding transcripts, polyadenylated long non-coding RNAs, polyadenylated anti-sense transcripts and a variety of non-polyadenylated RNAs such as microRNAs. Although the function of many of these transcripts is unknown, they are suggested to play a direct or regulatory role in the delicately orchestrated processes that ensure HCMV replication and life-long persistence. This review focuses on annotating the complete viral genome based on three sources of information. First, previous reviews were used as a template for the functional keywords to ensure continuity; second, the Uniprot database was used to further enrich the functional database; and finally, the literature was manually curated for novel functions of HCMV gene products. Novel discoveries were discussed in light of the viral life cycle. This functional annotation highlights still poorly understood regions of the genome but more importantly it can give insight in functional clusters and/or may be helpful in the analysis of future transcriptomics and proteomics studies.

## Introduction

Human cytomegalovirus (HCMV) is a common opportunistic pathogen with a worldwide prevalence of 45–100% depending on age, location, gender and socio-economic status (Cannon et al., 2010). Initial infection is followed by life-long persistence characterized by periodical reactivation episodes. Whereas in healthy individuals, initial infection and reactivation of the virus usually does not result in morbidity (Boeckh and Geballe, 2011); the virus can cause devastating complications in neonates and immune-compromised patients such as birth defects, systemic failure and rejection of the transplanted organ (Griffiths, 2006; Pass et al., 2006; Kenneson and Cannon, 2007; Crough and Khanna, 2009).

HCMV is a double-stranded DNA virus and with a genome length of 235 kB it has the largest genome of the Herpesvirus family. The genome contains a unique long (UL) and a unique short (US) region each flanked by terminal (TRL and TRS), and internal (IRL and IRS) inverted repeats (Murphy and Shenk, 2008). The genetic map of clinical strain Merlin was thought to contain 165 protein coding genes (Dolan et al., 2004). A later study identified an additional four new protein coding transcripts, i.e., RL8A, RL9A, UL150A, and US33A. In this study, it had already been suggested that more ORFs could exist which code for small proteins (Gatherer et al., 2011). A recent study confirmed this hypothesis and revealed a previously unprecedented complexity of HCMV gene expression when they identified an additional 604 protein coding ORFs, most of which were very short and located upstream of longer ORFs (Stern-Ginossar et al., 2012). In addition to protein coding genes, HCMV also produces polyadenylated non-coding RNAs. A first type of non-coding RNAs are the abundantly produced long non-coding RNA2.7, RNA1.2, RNA4.9, and RNA5.0 which do not overlap with protein coding regions. Secondly, non-coding RNAs are produced anti-sense of protein coding regions (Zhang et al., 2007; Gatherer et al., 2011). Finally, HCMV also codes for non-poly-adenylated RNAs, e.g., micro-RNAs which play a regulatory role (Dhuruvasan et al., 2011).

Expression of HCMV genes in permissive cell types proceeds in a temporal cascade initiated with the expression of immediate-early genes followed by the production of early, early-late and late transcripts (Wathen and Stinski, 1982; Stinski et al., 1983; Stenberg et al., 1985). In cell types that support the establishment of latency, such as CD14^+^ monocytes and CD34^+^ progenitor cells, an alternative transcription program is followed in which a limited set of transcripts sustains the latent state of the virus (Bevan et al., 1991; Beisser et al., 2001; Goodrum et al., 2002; Jenkins et al., 2004; Cheung et al., 2006; Goodrum et al., 2007; Reeves and Sinclair, 2009; Poole et al., 2013).

In a large scale study Dunn et al. (2003) identified 45 core gene products of which 78% are essential for growth in fibroblasts and appear to be conserved amongst all Herpesviruses, the remaining essential proteins are either HCMV or β-Herpesvirus specific. Equally interesting was the observation of 117 proteins which were dispensable for growth in fibroblasts. In addition, several genes appeared to be involved in viral growth suppression. UL9, UL20a, UL23, or US30 gene deletion resulted in enhanced growth in fibroblasts whereas similar observations were done for the UL10 and UL16 gene in endothelial cells and for the US16 and US19 gene in HMVECs. Although this appears counterproductive for viral replication, it was suggested that they may be part of a mechanism to prevent massive cellular damage and host death and/or to be involved in suppressing lytic infections to facilitate the establishment of latency. Largely confirming the gene deletion analysis of Dunn et al. (2003); Yu et al. (2003) performed random transposon mutations in HCMV and identified 41 essential, 88 non-essential, and 27 augmenting ORFs (Yu et al., 2003). However, at the time, the function of many of these genes was still unknown. Several of these gene products were later on functionally annotated by Mocarski (2007) in a comparative study between HCMV, HHV-6 and HHV-7 (Mocarski, 2007). This overview of HCMV gene functions dates from 2007 and in the meantime numerous gene products were investigated since. In addition, some gene products were implicated in new viral processes. For example, UL32 (pp150) was described to be involved in maturation during the lytic cycle (Aucoin et al., 2006). However, recently, a role for this gene product was also delineated in gene expression regulation and modulation of the host cell cycle (Bogdanow et al., 2013). In addition, numerous genes with a previously unknown function were investigated and are now attributed with a role in the viral life cycle. In this review, we provide an updated, non-exhaustive functional annotation of the HCMV genome based on curation of the literature, previous reviews and the Uniprot database (Supplementary Table 1).

## Methodology

The functional annotation presented here is based on three different sources of information. First, the functional keywords published in Dunn et al. (2003); Mocarski (2007) were included for each gene. Second, the ontology “biological process” found in Uniprot (Apweiler et al., 2004; Boutet et al., 2007) for each gene was used, even predicted annotations. If this information was not available, the ontology “cellular component” was used, the latter was intended to provide additional information on gene products, e.g., on the presence of a certain protein in the tegument or virion. Only reviewed Uniprot entries were included in the data presented here and, unless otherwise stated, the entry for the clinical strain Merlin was used. Finally, literature was manually searched using Pubmed (http://www.ncbi.nlm.nih.gov/pubmed, searches performed in December 2013) to fill in functional annotations discovered since the last overview in 2007. For each gene, the following searches were performed in Pubmed: “gene name and cytomegalovirus” and “gene name and HCMV.” Functional annotations were based on the information found in the title and/or abstract of retrieved publications. Each unique function was summarized in a keyword as others have done before (Dunn et al., 2003; Mocarski, 2007). Additionally, the time-kinetics of each gene product and whether or not the ORF is dispensable for growth in fibroblasts is provided based on a gene deletion study by Dunn et al. (2003); and a study using random transposon mutations in HCMV genes by Yu et al. (2003).

In addition to previous reviews (Dunn et al., 2003; Mocarski, 2007), we also included functional annotations for RL5A, RL8A, RL9A, UL1, UL37, UL81ast, UL118, UL131A, UL145 and all four lncRNAs. Recently, several genes (RL3, RL5, RL7, RL8, UL41, UL49.5, UL58, UL60, UL63, UL66, UL106, UL107, UL125, UL126, UL131, UL137, UL143, UL151, US4, US5, and US35) were shown not to code for proteins, these genes were omitted from this functional annotation (Davison et al., 2013).

## Functional annotation of HCMV genes during the lytic viral life cycle

Since the publications of Dunn et al. (2003) and Mocarski (2007), the functions of several genes have been unraveled. The aim of the section below is to review these novel functions in the context of the viral life cycle.

HCMV can infect a variety of cell types including, but not limited to, fibroblasts, endothelial cells, epithelial cells and cells of the myeloid lineage (Sinzger et al., 1999; Arrode and Davrinche, 2003; Durose et al., 2012; O'Connor and Shenk, 2012; Bayer et al., 2013). Depending on the cell type, the virus enters the cell by membrane fusion or by pH-mediated endocytosis. The former process is found in fibroblasts and mediated by the gH/gL/gO protein complex; the latter means of entry is typical for epithelial and endothelial cells and requires the gH/gL/UL128/UL130/UL131 pentameric complex (Wang and Shenk, 2005; Ryckman et al., 2008). Different proteins have been attributed a role in cell tropism or cell-specific replication before (Dunn et al., 2003; Mocarski, 2007). Recently, it was shown that the UL128-UL131A cluster rapidly mutates in fibroblasts and UL128 gene mutations appear to serve as a means to optimize viral replication in fibroblasts (Stanton et al., 2010). Based on mutational studies, UL131A was also found to be important for endothelial tropism as part of the pentameric entry complex (Schuessler et al., 2012). Besides the UL128-UL131 locus, US16 and UL78 (Mocarski, 2007; Bronzini et al., 2012; O'Connor and Shenk, 2012) and possibly RL4-RL5, RL13, UL1, UL39, UL83, UL109, UL110, UL111A, UL132, UL148, and US22 are involved in epithelial tropism (Stanton et al., 2010; Womack, 2011; Shikhagaie et al., 2012).

Following entry, a temporal cascade starts resulting in expression of immediate early (IE), early (E) and late (L) HCMV proteins (Supplementary Table 1). There is a general consensus that the production of IE1 and IE2, assisted by several additional proteins, initiates the viral replication (Colberg-Poley, 1996). In addition, US24 was suggested to play an important role in the progression of the cascade as the replication cycle of US24-deficient viruses is blocked after the viral DNA reaches the nucleus and before immediate-early mRNAs are transcribed (Feng et al., 2006).

During the progression of HCMV replication, the virus tightly modulates its own gene expression at various times during the temporal cascade. A good example of a complex regulatory mechanism which includes both viral and cellular factors is the control of the major immediate-early promotor (MIEP). The activity or repression of the MIEP is dependent on its association with active or inactive chromatin and thus with acetylated and demethylated histones or deacetylated and methylated histones, respectively (Reeves, 2011). Furthermore, the MIEP contains motifs for the binding of cellular and viral activating (e.g., CREB, NFkB, AP-1) and/or repressive transcription factors (e.g., SBP, modulator recognition factor, PDX1, YY1, methylated DNA-binding protein, GFI-1, ERF) (Meier and Stinski, 1996; Stinski and Meier, 2007). Two additional viral products, i.e., pp71 (UL82), a tegument protein; and pp65 (UL83), mostly known for its properties as an immune modulator, were found to transactivate or regulate the MIEP (Homer et al., 1999; Hofmann et al., 2002; Cristea et al., 2010; Arcangeletti et al., 2011; Tomtishen, 2012). Other proteins such as UL97, products of the UL28-UL29 locus and US28 mediate the MIEP via cellular transcription factors (i.e., US28), via HDACs (UL97 and UL28-UL29) or via modulation of the host (UL32/pp150) (Mitchell et al., 2009; Wen et al., 2009; Terhune et al., 2010; Bigley et al., 2013; Bogdanow et al., 2013). Also US3 gene expression is tightly regulated. The US3 gene is an auxillary IE gene which retains the MHC-I heavy chain proteins in the ER and works as an immunomodulator. The US3 gene is activated by pp71, IE1, IE2 and TRS1 while repression is mediated by UL34 (Biegalke, 1999; Mocarski, 2007). In addition, UL37.1/UL38 and UL84 inhibited IE1 and IE2-mediated US3 expression (Biegalke, 1999). Later in the viral life cycle during late gene expression, UL79, UL87 UL91, UL92, and UL95 were recently found to be involved in the accumulation of late genes (Isomura et al., 2011; Perng et al., 2011; Omoto and Mocarski, 2013, 2014).

During late stages of infection, the virus ensures that viral DNA replication is established (Tandon and Mocarski, 2012). Recently, UL36, UL49, UL80, UL100, UL117, and TRS1 have been attributed an essential role in DNA replication (Smith and Pari, 1995a; Krzyzaniak et al., 2007; Nguyen et al., 2008; Qian et al., 2008; Marshall et al., 2009; Zhang et al., 2010; Wang et al., 2013a) while the importance of UL112-UL113 and UL84 during DNA replication was further delineated (Colletti et al., 2007; Gao and Pari, 2009; Kagele et al., 2009; Kim and Ahn, 2010; Strang et al., 2012). In addition, UL76 was found to modulate the expression of the essential protein UL77 possibly as part of a mechanism to modulate UL77 levels required for efficient viral replication (Dunn et al., 2003; Isomura et al., 2010). Interestingly, UL55 and UL76 were identified to inhibit DNA replication suggesting that these viral factors are likely key proteins to fine-tune the replication process (Wang et al., 2004; Xiaofei et al., 2012).

Upon the creation of new genomes, the DNA is packaged in newly formed capsids. Based on predictions, UL80.5 and UL93 may be involved in capsid formation (Apweiler et al., 2004; Loveland et al., 2007) whilst the role of UL51 in packaging of the genome was confirmed. Interestingly, also the neighboring gene UL52 was attributed a role in genome packaging (Mocarski, 2007; Borst et al., 2008, 2013). After the encapsidation of the DNA, the immature virion is transported from the nucleus to the cytoplasm to undergo maturation processes such as primary and secondary envelopment (Tandon and Mocarski, 2012). Recently, the protein products of the UL133-UL138 region were found to be important for the formation of the cytoplasmic assembly complex, more specifically in endothelial cells (Bughio et al., 2013). Further, UL96 has been found to stabilize pp150-associated nucleocapsids during translocation from the nucleus to the cytoplasm (Tandon and Mocarski, 2011) and UL32/pp150 preserves the integrity of the immature virion during maturation events. Also, UL32/pp150 enables the proper assemby of the tegument layers during the final phase of maturation (Aucoin et al., 2006). Additional mutation or deletion studies showed crucial roles for UL71, UL74, UL94, and US17 in the final stages of secondary envelopment (Jiang et al., 2008; Schauflinger et al., 2011; Meissner et al., 2012; Phillips and Bresnahan, 2012; Gurczynski et al., 2014). US28 was predicted, based on homology, to also have a role in maturation; however, further research will need to validate this hypothesis. UL89 suppression using shRNA leads to a defect in viral particle formation although a specific mechanism has not been identified (Thoma and Bogner, 2010). Finally, UL74, US17, and UL103 were attributed a role in the egress of newly formed virions (Jiang et al., 2008; Thoma and Bogner, 2010; Ahlqvist and Mocarski, 2011).

Throughout the life cycle, viral proteins which regulate cellular trafficking are integral in the viral replication cycle. Upon membrane fusion, the capsid is released in the cytoplasm and transported to the nuclear pore to release the viral DNA in the nucleus. After replication, new capsids are transported to the cytoplasm to acquire a host-derived membrane before virion release (Fulcher and Jans, 2011; Henaff et al., 2012). As part of this process, a role in directing capsids to the final sites of envelopment has been proposed for UL32/pp150 (Kalejta, 2008). Whilst UL52 has been proposed to be involved in capsid transport, UL50 and UL53 have been confirmed as factors responsible for nuclear egress for which they recruit UL97 (Mocarski, 2007; Sharma et al., 2014).

The composition of the virion depends on the nature of the starting cell line and methods of culturing and purification (Gibson, 2008b). However, it consists of highly conserved proteins categorized into virion capsid components, tegument and envelope proteins (Dunn et al., 2003; Mocarski, 2007; Gibson, 2008a; Kalejta, 2008; Tomtishen, 2012). Several new virion proteins have been identified recently, some still with an unknown function. The UL1 protein, a late protein, is now determined to be an envelope protein and was found at assembly sites in the presence of other viral structural proteins (Shikhagaie et al., 2012). Analysis of the UL1 sequence positions the gene in the RL11 family which also includes RL5A, RL6, RL11, RL12, RL13, and UL4-UL14 genes (Mocarski, 2007; Shikhagaie et al., 2012). Interestingly, but not surprising, as the UL1 gene is thought to originate from a RL11-RL13 gene duplication, is that the RL13 gene product has also been described as a new glycosylated envelope protein (Stanton et al., 2010). Although not experimentally verified, Uniprot suggests (prediction based on similarity) that also RL12 and UL29 may be a part of the virion (Apweiler et al., 2004). Finally UL26 has been attributed a role in virion stability (Kalejta, 2008).

The progression of the viral life cycle also depends on the capability of the virus to modulate host cell processes such as cellular gene expression and apoptosis to its advantage. Recently, US27 was identified as a modulator of cellular gene expression (Arnolds et al., 2013) whilst UL28-UL29, UL38, UL79 and RNA2.7 were found to control apoptosis (Reeves et al., 2007; Terhune et al., 2007; Moorman et al., 2008; Siew et al., 2009; Xuan et al., 2009; Qian et al., 2011; Fliss and Brune, 2012; Costa et al., 2013; Savaryn et al., 2013).

## Novel functional annotations of HCMV genes expressed during latency

As all herpesviruses, HCMV establishes latency but HCMV specifically resides in myeloid cells such as CD34^+^ progenitor cells and CD14^+^ monocytes (Taylor-Wiedeman et al., 1991; Mendelson et al., 1996; Sindre et al., 1996; Bolovan-Fritts et al., 1999). Upon differentiation stimuli, latent virus can reactivate and re-enter the lytic life cycle resulting in the production of infectious virions (Soderberg-Naucler et al., 1997; Zhuravskaya et al., 1997; Hahn et al., 1998; Soderberg-Naucler et al., 2001; Goodrum et al., 2002; Reeves et al., 2005; Huang et al., 2012). During latency, HCMV remains present as an episome and produces a limited set of transcripts (Bolovan-Fritts et al., 1999; Goodrum et al., 2002; Bego et al., 2005; Cheung et al., 2006; Goodrum et al., 2007; Petrucelli et al., 2009; Avdic et al., 2011; Bego et al., 2011; Keyes et al., 2012; Poole et al., 2013; Rossetto et al., 2013).

After the identification of the first putative latency transcripts (Kondo and Mocarski, 1995; Kondo et al., 1996), several large scale studies have been set up to elucidate the latency-associated transcriptome. Upon infection of non-permissive cells, transcripts from 65 different loci have been observed which, over time, lead to robust expression of a discrete set of transcripts (Goodrum et al., 2002; Cheung et al., 2006; Rossetto et al., 2013). Noteworthy is that several of these identified transcripts, i.e., RL3, RL5, RL7, UL41, UL66, and UL131 were recently described not to code for proteins (Davison et al., 2013). Of the remaining transcripts, 30 were found in only one study by Goodrum et al. (2002), whilst UL28, UL37, UL50, UL52, UL95, UL114, UL123, UL126A, US27, RNA2.7, and RNA4.9 were found uniquely in another by Rossetto et al. (2013). Further studies also pinpointed UL82/pp71 and UL128 as possible latency-associated transcripts (LATs) (Cheung et al., 2006; Penkert and Kalejta, 2012).

A number of transcripts were found in more than one study, i.e., RL7, UL3, UL67, UL68, UL84, UL87, UL108, UL110, UL133, UL135, US28 (Beisser et al., 2002; Goodrum et al., 2002; Cheung et al., 2006; Keyes et al., 2012; Rossetto et al., 2013) but thus far only UL81ast/LUNA (Goodrum et al., 2002; Bego et al., 2005, 2011; Keyes et al., 2012; Rossetto et al., 2013), UL111A (Cheung et al., 2006; Avdic et al., 2011; Rossetto et al., 2013), UL138 (Goodrum et al., 2002, 2007; Petrucelli et al., 2009; Rossetto et al., 2013) and, depending on the virus used and the presence of GATA transcription binding sites, UL144 (Poole et al., 2013) were extensively characterized as LATs. Further research will show the validity of other potential LATs and dive deeper into the actual function of these transcripts and possibly their gene products during HCMV latency.

## New genes involved in immunomodulation

HCMV modulates both the host's innate and adaptive immunity though various pathways (Jackson et al., 2011; Noriega et al., 2012a). In fact, in our functional annotation, 14 HCMV gene products were attributed a new role, or a proposed role, in immunomodulation whilst the involvement of 19 genes in immunomodulation were further expanded or confirmed (Supplementary Table 1).

HCMV is well known to interfere with antigen presentation on MHC-I molecules thereby evading T-cell and NK-regulated immunity (Jackson et al., 2011; Noriega et al., 2012a). Since the last reviews (Dunn et al., 2003; Mocarski, 2007), the roles of HCMV products in modulation of MHC-I expression (US2, US3, US10, US11, UL82/pp71), interference in antigen prestentation (US6) or their debilitating capacity on T-cell or NK cell recognition and function (UL16, UL18, UL141, UL142, US2) were further characterized (Wiertz et al., 1996; Cosman et al., 2001; Odeberg et al., 2003; Wills et al., 2005; Oresic et al., 2006; Dugan and Hewitt, 2008; Kim et al., 2008; Oresic and Tortorella, 2008; Ashiru et al., 2009; Muller et al., 2010; Park et al., 2010; Prod'homme et al., 2010; Noriega et al., 2012b; Penkert and Kalejta, 2012; Hesse et al., 2013; Smith et al., 2013). In addition, several gene products have a new role in immunomodulation. UL37.3 was found to be an MHC-I-like molecule encoded by the virus (Wyrwicz and Rychlewski, 2008; Revilleza et al., 2011). Alternatively, UL49.5 was found to influence MHC-I recognition by T-cells while UL11 caused functional paralysis of T-cells (Oosten et al., 2007; Gabaev et al., 2011).

HCMV also expresses an array of cytokine- and chemokine-like molecules (McSharry et al., 2012). The involvement of UL144, UL146, UL147, and US28 in cytokine- and chemokine-mediated processes was already reported before; but several groups further delineated their function (Poole et al., 2008; Stropes et al., 2009; Luttichau, 2010). Also, it was shown that UL33 and UL78 form heteromers with CCR5 and CXCR4 chemokine receptors resulting in a predominantly negative effect on CCR5 and CXCR4 functions and expression; without altering the chemokine binding properties of both receptors (Tadagaki et al., 2012). Alternatively, UL7 was found to be involved in modulating cytokine expression in DCs and myleoid cell lines. Through a SLAM domain UL7 can also mediate cellular adhesion to monocyte-derived DCs (Engel et al., 2011). In addition, UL22A has been suggested to play a role in infected DCs (Raftery et al., 2009). Further, UL128 has been shown to modulate cytokine expression to induce PBMC proliferation (Zheng et al., 2012). Also US17 was recently implicated in modulation of host pathways by controlling the virion so that it elicits a balanced immune response (Gurczynski et al., 2014). For UL138 an apparently contradictory role has been described. UL138, a known LAT (Goodrum et al., 2007), upregulates TNFR surface expression and potentiates the action of TNFα, two processes much associated with pro-inflammatory responses (Chu, 2013). However, TNFα has been attributed reactivating properties and thus it is postulated that UL138 may sensitize latently infected cells to TNFα-mediated reactivation of HCMV. Finally UL139 is proposed to have an immunomodulatory role due to its homology to CD24 whilst RL12 and RL13 are likely involved in limiting host antibodies through their IgG binding capacity (Qi et al., 2006; Cortese et al., 2012). Other genes such as US7, US9 and TRS have been predicted to be involved in immunomodulation (Apweiler et al., 2004).

## Conclusion

The large genome of HCMV is expressed in a complex and tightly regulated temporal cascade. Numerous protein products of the virus are cis- and trans-acting factors which modulate viral and cellular gene expression thereby ensuring maximal efficiency of the viral replication whilst carefully minimalizing disruptive cellular processes such as apoptosis and immune defense. In this review, we focussed on updating the functional annotation of the complete HCMV genome. Functional annotations such as the one presented here, and previously published annotations (Dunn et al., 2003; Mocarski, 2007), can be an aid in transcriptome or proteomics studies. This functional annotation is useful to look at gene clusters with similar functions, to perform functional pathway analysis or may suggest future research goals to investigate if neighboring genes with an unknown function possibly fit the same annotation as the rest of the cluster. Continues efforts of various groups have provided a functional annotation of most of the HCMV gene products. However, the representation of several functional categories stand out. First of all, the large number of genes associated with latency is striking. While only four LATs have been extensively characterized, many more transcripts were found in high-throughput studies. The challenge lies in identifying protein products for these transcripts and to determine their true role during HCMV latency and reactivation. In addition, most of the recent findings reveal that many HCMV gene products are involved in immunomodulation. This is not unusual for a virus which persists for life but only now we are beginning to understand the true complexity of the various cellular pathways HCMV modulates. Further research is vital to compose the complete picture of all immunomodulatory pathways which HCMV uses to enable lytic and latent infection. It is interesting in this regard to investigate gene clusters, e.g., UL141-UL148 in which some genes products have been attributed a function in immunomodulation, e.g., UL141, UL142, UL144, UL146, UL147 whilst others (UL145, UL148, UL149, and UL150) have an unknown function. Future studies investigating these regions will provide information if currently unknown genes also play a role in immunomodulation.

The current table is based on the gene map of genomics and transcriptomics studies (Dunn et al., 2003; Gatherer et al., 2011). However, recently it was revealed that the coding capacity of HCMV is far greater than originally assumed. Stern-Ginossar et al. (2012) reported no less than 751 translated ORFs and 53 novel proteins originating from ORFs not overlapping the known ORFs. As currently the function of these genes and proteins is unknown, further reductionist studies are required to determine their role in the viral life cycle.

## Author contributions

Ellen Van Damme and Marnix Van Loock gathered and combined the information to functionally annotate the HCMV genome, and wrote the manuscript describing novel functions of HCMV genes during the viral life cycle.

### Conflict of interest statement

The authors declare that the research was conducted in the absence of any commercial or financial relationships that could be construed as a potential conflict of interest.
