# Supplementary material for: Functional annotation of human cytomegalovirus gene products: an update
Source: Front Microbiol. 2014 May 19;5:218. doi: 10.3389/fmicb.2014.00218 (PMC4032930; doi:10.3389/fmicb.2014.00218)
Supplement: Supplementary file 1 [file DataSheet1.pdf]

Gene	Growth	Kinetics	Uniprot entry	Function keyword	Process/protein/gene family	Reference
RL1	D[a,b]	N/A	Q6SWD5	Unknown		
RL2	D[a,b]	E (Chambers et al., 1999)	N/A	Latency*		(Goodrum et al., 2002)
RL4	D[a,b]	E (Mocarski Jr, 1996;Chambers et al., 1999;Dunn et al., 2003)	N/A	Cell tropism/Cell type-specific replication*	May be involved in cell tropism epithelial cells	(Womack, 2011)
RL4	D[a,b]	E (Mocarski Jr, 1996;Chambers et al., 1999;Dunn et al., 2003)	N/A	Latency*		(Goodrum et al., 2002)
RL5A	N/A	E (Hutchinson et al., 1986)	F5HF23	Unknown	RL11 family†	
RL6	D[a,b]	L (Chambers et al., 1999); E/L (Hutchinson et al., 1986)	Q6SWD3	Latency*	RL11 family†	(Goodrum et al., 2002)
RL8A	N/A	N/A	F7V995	Unknown		
RL9A	N/A	N/A	F7V996	Unknown		
RL9	D[a,b]	L (Chambers et al., 1999)	N/A	Unknown		
RL10	D[a,b]	E/L (Chambers et al., 1999)	F5HI32	Virion protein	Envelope protein† Glycoprotein	(Apweiler et al., 2004;Mocarski Jr, 2007)
RL11	D[a,b]	L (Chambers et al., 1999)	Q6SWD1	Immunomodulation*	IgG Fc-binding capacity† Glycoprotein† RL11 family†	(Mocarski Jr, 2007)
RL12	D[a,b]	E/L (Chambers et al., 1999)	Q6SWD0	Virion protein*	Putative membrane glycoprotein† RL11 family†	(Apweiler et al., 2004;Mocarski Jr, 2007)
RL12	D[a,b]	E/L (Chambers et	Q6SWD0	Immunomodulation*	IgG Fc-binding	(Cortese et al.,

		al., 1999)			capacity † Putative membrane glycoprotein † RL11 family †	2012)
RL13/TRL14	D[a,b]	E/L (Chambers et al., 1999)	Q6SWC9	Cell tropism/Cell type-specific replication*	May be involved in cell tropism Glycoprotein RL11 family †	(Stanton et al., 2010)
RL13/TRL14	D[a,b]	E/L (Chambers et al., 1999)	Q6SWC9	Virion protein	Glycoprotein	(Apweiler et al., 2004;Stanton et al., 2010)
RL13/TRL14	D[a,b]	E/L (Chambers et al., 1999)	Q6SWC9	Immunomodulation*	IgG Fc-binding glycoprotein	(Cortese et al., 2012)
RL13/TRL14	D[a,b]	E/L (Chambers et al., 1999)	Q6SWC9	(DNA) Replication	RL13 repressed replication in multiple cell types Glycoprotein	(Stanton et al., 2010)
UL1	D[b]	E/L (Chambers et al., 1999)	Q6SWC8	Virion protein	Glycoprotein RL11 family †	(Apweiler et al., 2004)
UL1	D[b]	E/L (Chambers et al., 1999)	Q6SWC8	Cell tropism/Cell type-specific replication*	May be involved in cell tropism in epithelial cells Glycoprotein RL11 family †	(Shikhagaie et al., 2012)
UL1	D[b]	E/L (Chambers et al., 1999)	Q6SWC8	Assembly/Maturation/Egress*	Localized at the cytoplasmic site of virion assembly and secondary envelopment Glycoprotein	(Shikhagaie et al., 2012)
UL2	MGD[a]/ D[b]	L (Chambers et al., 1999)	Q6SWC7	Unknown	Putative membrane protein †	
UL3	D[a,b]	L (Chambers et al., 1999)	A8T755	Unknown		

UL4	D[a,b]	E (Mocarski Jr, 1996;Chambers et al., 1999)	Q6SWC6	Virion protein	Glycoprotein† RL11 family†	(Chang et al., 1989;Apweiler et al., 2004)
UL4	D[a,b]	E (Mocarski Jr, 1996;Chambers et al., 1999)	Q6SWC6	Latency*	Glycoprotein† RL11 family†	(Goodrum et al., 2002)
UL5	D[a,b]	E (Chambers et al., 1999)	Q6SWC5	Virion protein	Virion membrane protein?† RL11 family†	(Mocarski Jr, 2007)
UL5	D[a,b]	E (Chambers et al., 1999)	Q6SWC5	Latency*	Virion membrane protein?† RL11 family†	(Goodrum et al., 2002)
UL6	D[a,b]	N/A	Q6SWC4	Unknown	Putative membrane glycoprotein† RL11 family†	
UL7	D[a,b]	L (Chambers et al., 1999)	Q6SWC3	Immunomodulation	Modulation chemo- and/or cytokines Mediation immune cell adhesion Glycoprotein RL11 family†	(Apweiler et al., 2004;Engel et al., 2011)
UL8	D[a,b]	N/A	Q6SWC2	Unknown	Putative membrane glycoprotein† RL11 family†	
UL9	EG[a]/D[b]	L (Chambers et al., 1999)	F5H9T4	Viral growth	Temperance in fibroblasts† Putative membrane glycoprotein† RL11 family†	(Mocarski Jr, 2007)
UL10	D[a,b]	N/A	Q6SWC0	Viral growth	Temperance in RPE† Putative membrane glycoprotein† RL11 family†	(Mocarski Jr, 2007)

UL11	MGD[a]/ D[b]	E (Hitomi et al., 1997)	Q6SWB9	Immunomodulation	Modulation T-cell signaling/function Membrane glycoprotein † RL11 family †	(Gabaev et al., 2011)
UL12	MGD[a]/ D[b]	N/A	A8T769	Unknown		
UL13	D[a,b]	E (Chambers et al., 1999); IE/L (Wang et al., 2013b)	Q6SWB8	Unknown	Putative secreted protein †	
UL14	MGD[a]/ D[b]	L (Chambers et al., 1999)	Q6SWB7	Unknown	Putative membrane glycoprotein † UL14 family †	
UL15A	D[b]	N/A	F5HAE6	Unknown	Putative membrane protein †	
UL15	D[a]	L (Chambers et al., 1999)	N/A	Unknown		
UL16	D[a,b]	E (Kaye et al., 1992)	F5HG68	Immunomodulation	Modulation NK cell signaling/function Membrane glycoprotein †	(Cosman et al., 2001;Dunn et al., 2003;Odeberg et al., 2003;Mocarski Jr, 2007;Muller et al., 2010)
UL16	D[a,b]	E (Kaye et al., 1992)	F5HG68	Viral growth	Temperance in fibroblasts †	(Mocarski Jr, 2007)
UL17	D[a,b]	E (Chambers et al., 1999)	F5HHT4	Latency*		(Goodrum et al., 2002)

UL18	D[a,b]	L (Hassan-Walker et al., 1998;Chambers et al., 1999)	F5HFB4	Immunomodulation	MHC-I homologue Modulation NK cell signaling/function Putative membrane glycoprotein† UL18 family†	(Chapman et al., 1999;Vitale et al., 1999;Dunn et al., 2003;Apweiler et al., 2004;Mocarski Jr, 2007;Kim et al., 2008)
UL19	D[a,b]	N/A	F5HI68	Unknown		
UL20	MGD[a]/D[b]	E (Jelcic et al., 2011)	F5H9Z4	Immunomodulation*	Modulation T-cell signaling/function*	(Beck and Barrell, 1991;Mocarski Jr, 2007)
UL21	SGD[a]	L (Chambers et al., 1999)	P16759 (AD169)	Unknown		
UL21.5/UL21A	EG[a]/GD[b]	E (Fehr and Yu, 2010)	F5HH39	Modulation of host cell cycle/protein synthesis	Degradation of cyclin A (primate CMV)	(Apweiler et al., 2004;Caffarelli et al., 2013)
UL21.5/UL21A	EG[a]/GD[b]	E (Fehr and Yu, 2010)	F5HH39	Viral growth	Temperance in fibroblasts†	(Mocarski Jr, 2007)
UL21.5/UL21A	EG[a]/GD[b]	E (Fehr and Yu, 2010)	F5HH39	(DNA) replication	Facilitates DNA synthesis	(Mocarski Jr, 2007;Fehr and Yu, 2010; 2011)
UL21.5/UL21A	EG[a]/GD[b]	E/L (Boriskin and Butcher, 2001)	F5HF91	Immunomodulation	Modulation chemo- and/or cytokines	(Mocarski Jr, 2007)
UL22A	N/A	N/A	F5HF90	Immunomodulation*	Possible role on DCs Secreted glycoprotein†	(Raftery et al., 2009)
UL22A	N/A	N/A	F5HF90	Virion protein	Secreted glycoprotein†	(Apweiler et al., 2004;Mocarski Jr, 2007)
UL22	N/A	L (Andre et al., 1999)	N/A	Unknown		
UL23	EG[a]/D[b]	E/L (Adair et al., 2002)	F5HDM3	Viral growth	Temperance in fibroblasts† Tegument protein† US22 family†	(Mocarski Jr, 2007)

UL23	EG[a]/D[b]	E/L (Adair et al., 2002)	F5HDM3	Virion protein	Tegument protein † US22 family †	(Dunn et al., 2003;Apweiler et al., 2004;Mocarski Jr, 2007)
UL24	D[a,b]	E/L (Adair et al., 2002)	F5H9N4	Virion protein	Tegument protein † US22 family †	(Dunn et al., 2003;Apweiler et al., 2004;Mocarski Jr, 2007)
UL24	D[a,b]	E/L (Adair et al., 2002)	F5H9N4	Cell tropism/Cell type-specific replication	Involved in cell tropism in endothelial cells Tegument protein † US22 family †	(Dunn et al., 2003;Mocarski Jr, 2007)
UL25	D[a,b]	L (Baldick and Shenk, 1996;Battista et al., 1999)	F5HGJ4	Virion protein	Tegument phospoprotein † UL25 family †	(Baldick and Shenk, 1996;Dunn et al., 2003;Apweiler et al., 2004;Mocarski Jr, 2007)
UL26	SGD[a]/ GD[b]	E (Chambers et al., 1999); IE/E (Stamminger et al., 2002)	F5HGG3	Virion protein	Tegument protein † US22 family †	(Baldick and Shenk, 1996;Dunn et al., 2003;Apweiler et al., 2004;Mocarski Jr, 2007)
UL26	SGD[a]/ GD[b]	E (Chambers et al., 1999); IE/E (Stamminger et al., 2002)	F5HGG3	Gene expression/regulation	Activator of MIEP † Tegument protein † US22 family †	(Baldick and Shenk, 1996;Dunn et al., 2003;Mocarski Jr, 2007)
UL26	SGD[a]/ GD[b]	E (Chambers et al., 1999); IE/E (Stamminger et al., 2002)	F5HGG3	Virion stability		(Kalejta, 2008)
UL27	D[a]/GD[b]	E (Chambers et al., 1999)	Q6SWA4	Unknown	Maribavir resistance †	

UL28	SGD[a]/ GD[b]	E (Mitchell et al., 2009)	P16847	Gene expression/regulation	Activator of MIEP (via NuRD complex) US22 family[†]	(Mitchell et al., 2009;Terhune et al., 2010)
UL28	SGD[a]/ GD[b]	E (Mitchell et al., 2009)	P16847	Apoptosis	Regulation of p53 US22 family[†]	(Savaryn et al., 2013)
UL28	SGD[a]/ GD[b]	E (Mitchell et al., 2009)	P16847	Latency*	US22 family[†]	(Rossetto et al., 2013)
UL29	MGD[a]/ GD[b]	L (Chambers et al., 1999); E (Mitchell et al., 2009)	Q6SWA3	Virion protein*	US22 family[†]	(Apweiler et al., 2004)
UL29	MGD[a]/ GD[b]	L (Chambers et al., 1999); E (Mitchell et al., 2009)	Q6SWA3	Gene expression/regulation	Activator of MIEP (via NuRD complex) US22 family[†]	(Mitchell et al., 2009;Terhune et al., 2010)
UL29	MGD[a]/ GD[b]	L (Chambers et al., 1999); E (Mitchell et al., 2009)	Q6SWA3	Apoptosis	Regulation of p53 US22 family[†]	(Savaryn et al., 2013)
UL29	MGD[a]/ GD[b]	L (Chambers et al., 1999); E (Mitchell et al., 2009)	Q6SWA3	Cell tropism/Cell type-specific replication	Involved in cell tropism in endothelial cells[†] US22 family[†]	(Mocarski Jr, 2007;Savaryn et al., 2013)
UL30	SGD[a]/ GD[b]	L (Ma et al., 2014)	F5HGC2	Latency*		(Goodrum et al., 2002)
UL31	MGD[a]/ D[b]	L (Chambers et al., 1999; Ma et al., 2014)	Q6SWA0	Gene expression/regulation	Phosphoprotein	(Chang and Roizman, 1993;Dunn et al., 2003)
UL32 (pp150)	E[a,b]	L (Mocarski Jr, 1996;Chambers et al., 1999)	Q6SW99	Virion protein	Major tegument protein [†]	(Dunn et al., 2003;Apweiler et al., 2004;Mocarski Jr, 2007;Kalejta, 2008)

UL32 (pp150)	E[a,b]	L (Mocarski Jr, 1996;Chambers et al., 1999)	Q6SW99	Cellular trafficking	Transport capsid to nucleus post entry<br>Major tegument protein †	(Mocarski Jr, 2007;Kalejta, 2008)
UL32 (pp150)	E[a,b]	L (Mocarski Jr, 1996;Chambers et al., 1999)	Q6SW99	Assembly/Maturation/Egress	Final envelopment/egress<br>Major tegument protein †	(AuCoin et al., 2006)
UL32 (pp150)	E[a,b]	L (Mocarski Jr, 1996;Chambers et al., 1999)	Q6SW99	Gene expression/regulation	Regulator of IE gene expression<br>Major tegument protein †	(Bogdanow et al., 2013)
UL32 (pp150)	E[a,b]	L (Mocarski Jr, 1996;Chambers et al., 1999)	Q6SW99	Modulation of host cell cycle/protein synthesis	Sensor of the host cell cycle and differentiation state<br>Major tegument protein †	(Bogdanow et al., 2013)
UL32 (pp150)	E[a,b]	L (Mocarski Jr, 1996;Chambers et al., 1999)	Q6SW99	Latency*	Major tegument protein †	(Goodrum et al., 2002)
UL33 (UL33.1 & UL33.2	D[a,b]	E (Chambers et al., 1999); L (Mocarski Jr, 1996; Tadagaki et al., 2012)	Q6SW98	Immunomodulation	Modulation of chemo- and/or cytokine receptor through binding (CCR5/CXCR4)<br>Envelope protein †<br>GPCR-7TM family †	(Apweiler et al., 2004;Tadagaki et al., 2012)
UL33 (UL33.1 & UL33.2	D[a,b]	E (Chambers et al., 1999); L (Mocarski Jr, 1996; Tadagaki et al., 2012)	Q6SW98	Virion protein	Envelope protein †<br>GPCR-7TM family †	(Mocarski Jr, 2007)

UL33 (UL33.1 & UL33.2	D[a,b]	E (Chambers et al., 1999); L (Mocarski Jr, 1996; Tadagaki et al., 2012)	Q6SW98	Modulation of host cell cycle/protein synthesis	Envelope protein† GPCR-7TM family†	(Mocarski Jr, 2007)
UL34	E[a,b]	E/L (Chambers et al., 1999); E and L (2 proteins) (Biegalke et al., 2004)	F5HC16	Gene expression/regulation	US3 regulator (repressor)†	(Mocarski Jr, 2007)
UL35	MGD[a]/ D[b]	E (Chambers et al., 1999); E and L (2 proteins)(Liu and Biegalke, 2002)	F5HE12	Gene expression/regulation*	Possible regulator of IE gene expression Tegument phosphoprotein† UL25 family†	(Dunn et al., 2003;Mocarski Jr, 2007;Kalejta, 2008)
UL35	MGD[a]/ D[b]	E (Chambers et al., 1999); E and L (2 proteins)(Liu and Biegalke, 2002)	F5HE12	(DNA) Replication	Tegument phosphoprotein† UL25 family†	(Mocarski Jr, 2007)
UL35	MGD[a]/ D[b]	E (Chambers et al., 1999); E and L (2 proteins)(Liu and Biegalke, 2002)	F5HE12	Nucleotide repair/modification	DNA damage response Tegument phosphoprotein† UL25 family†	(Salsman et al., 2012)
UL35	MGD[a]/ D[b]	E (Chambers et al., 1999); E and L (2 proteins)(Liu and Biegalke, 2002)	F5HE12	Assembly/Maturation/Egress	Tegument assembly/viral egress Tegument phosphoprotein† UL25 family†	(Kalejta, 2008)

UL35	MGD[a]/ D[b]	E (Chambers et al., 1999); E and L (2 proteins)(Liu and Biegalke, 2002)	F5HE12	Virion protein	Tegument phosphoprotein † UL25 family †	(Dunn et al., 2003;Apweiler et al., 2004;Mocarski Jr, 2007)
UL36	D[a,b]	E (Chambers et al., 1999); IE (Colberg-Poley et al., 1992;Mocarski Jr, 1996)	F5HAY6	Apoptosis	Inhibitor of caspase-8 † IE tegument protein † US22 family †	(Dunn et al., 2003;Apweiler et al., 2004;Mocarski Jr, 2007;McCormick et al., 2010)
UL36	D[a,b]	E (Chambers et al., 1999); IE (Colberg-Poley et al., 1992;Mocarski Jr, 1996)	F5HAY6	(DNA) Replication	Initiation replication IE tegument protein † US22 family †	(Smith and Pari, 1995a)
UL36	D[a,b]	E (Chambers et al., 1999); IE (Colberg-Poley et al., 1992;Mocarski Jr, 1996)	F5HAY6	Gene expression/regulation	Alters cellular and viral gene expression IE tegument protein † US22 family †	(Colberg-Poley et al., 1992)
UL36	D[a,b]	E (Chambers et al., 1999); IE (Colberg-Poley et al., 1992;Mocarski Jr, 1996)		Virion protein	IE tegument protein † US22 family †	(Mocarski Jr, 2007)
UL37	D[b]	IE (Uniprot)	Q6SW94	Latency*		(Rossetto et al., 2013)
UL37.1	E[a]	IE (Mocarski Jr, 1996)	N/A	(DNA) Replication	Initiation replication	(Colberg-Poley et al., 1992;Smith and Pari, 1995a)

UL37.1	E[a]	IE (Mocarski Jr, 1996)	N/A	Apoptosis	vMIA †	(Dunn et al., 2003;Mocarski Jr, 2007)
UL37.1	E[a]	IE (Mocarski Jr, 1996)	N/A	Gene expression/regulation	US3 regulator	(Biegalke, 1999)
UL37.3	D[a]	IE (Mocarski Jr, 1996)	Q6SW94	(DNA) Replication	Initiation replication Glycoprotein †	(Colberg-Poley et al., 1992)
UL37.3	D[a]	IE (Mocarski Jr, 1996)	Q6SW94	Immunomodulation	MHC-I homologue Glycoprotein †	(Wyrwicz and Rychlewski, 2008)
UL37.3	D[a]	IE (Mocarski Jr, 1996)	Q6SW94	Viral growth	Glycoprotein †	(Hayajneh et al., 2001)
UL38	MGD[a]/ GD[b]	IE (Colberg-Poley et al., 1992;Mocarski Jr, 1996)	F5HG98	Latency*	Glycoprotein †	(Goodrum et al., 2002;Rossetto et al., 2013)
UL38	MGD[a]/ GD[b]	IE (Colberg-Poley et al., 1992;Mocarski Jr, 1996)	F5HG98	Apoptosis	Inhibitor of apoptosis Glycoprotein †	(Terhune et al., 2007;Moorman et al., 2008;Xuan et al., 2009;Qian et al., 2011)
UL38	MGD[a]/ GD[b]	IE (Colberg-Poley et al., 1992;Mocarski Jr, 1996)	F5HG98	Virion protein	Glycoprotein †	(Mocarski Jr, 2007)
UL38	MGD[a]/ GD[b]	IE (Colberg-Poley et al., 1992;Mocarski Jr, 1996)	F5HG98	Gene expression/regulation	US3 regulator (repressor) Glycoprotein †	(Biegalke, 1999)
UL39	D[a]	N/A	P16813 (AD169)	Cell tropism/Cell type-specific replication*	May be involved in cell tropism epithelial cells	(Womack, 2011)
UL39	D[a]	N/A	P16813 (AD169)	Latency*		(Goodrum et al., 2002)

UL40	D[b]	E/L (Chambers et al., 1999)	Q6SW92	Immunomodulation	Modulation NK cell signaling/function † Membrane glycoprotein †	(Apweiler et al., 2004;Mocarski Jr, 2007)
UL40	D[b]	E/L (Chambers et al., 1999)	Q6SW92	Latency*	Membrane glycoprotein †	(Goodrum et al., 2002)
UL41A	D[b]	N/A	F5HFG3	Virion protein	Membrane protein †	(Apweiler et al., 2004;Mocarski Jr, 2007)
UL42	D[a,b]	N/A	F5HHZ3	Unknown	Putative membrane protein †	
UL43	D[a,b]	L (Chambers et al., 1999;Adair et al., 2002)	Q6SW89	Virion protein	Tegument protein † US22 family †	(Dunn et al., 2003;Apweiler et al., 2004;Mocarski Jr, 2007)
UL44	E[a,b]	E/L (Mocarski Jr, 1996;Chambers et al., 1999); E (Iwayama et al., 1994)		Modulation of host cell cycle/protein synthesis	P53 repression	(Kwon et al., 2012)
UL44	E[a,b]	E/L (Mocarski Jr, 1996;Chambers et al., 1999); E (Iwayama et al., 1994)	F5HC97	(DNA) Replication	DNA polymerase progressivity subunit †	(Dunn et al., 2003;Apweiler et al., 2004;Mocarski Jr, 2007)
UL44	E[a,b]	E/L (Mocarski Jr, 1996;Chambers et al., 1999); E (Iwayama et al., 1994)	F5HC97	Latency*		(Goodrum et al., 2002;Rossetto et al., 2013)

UL45	D[a,b]	L (Patrone et al., 2003)	Q6SW87	(DNA) Replication	Ribonucleotide reductase homologue (enzymatically inactive) † Tegument protein †	(Dunn et al., 2003;Apweiler et al., 2004;Mocarski Jr, 2007)
UL45	D[a,b]	L (Patrone et al., 2003)	Q6SW87	Viral growth	Influence on viral growth at low MOI Tegument protein †	(Mocarski Jr, 2007)
UL45	D[a,b]	L (Patrone et al., 2003)	Q6SW87	Virion Protein	Tegument protein †	(Mocarski Jr, 2007)
UL46	E[a,b]	E/L (Chambers et al., 1999)	F5HA93	Virion protein	Minor capsid protein †	(Dunn et al., 2003;Apweiler et al., 2004;Mocarski Jr, 2007)
UL47	MGD[a]/ GD[b]	E/L (Chambers et al., 1999); L (Hyun et al., 1999)	Q6SW85	Virion protein	Tegument protein †	(Mocarski Jr, 2007)
UL47	MGD[a]/ GD[b]	E/L (Chambers et al., 1999); L (Hyun et al., 1999)	Q6SW85	Assembly/Maturation/Egress*	Tegument protein †	(Apweiler et al., 2004)
UL47	MGD[a]/ GD[b]	E/L (Chambers et al., 1999); L (Hyun et al., 1999)	Q6SW85	Cellular Trafficking*	May be involved in release of vDNA post entry Tegument protein †	(Mocarski Jr, 2007)
UL48	E[a]/GD[b]	L (Chambers et al., 1999)	Q6SW84	Viral growth	DUB activity contributes to viral growth (largest) Tegument protein †	(Apweiler et al., 2004;Kim et al., 2009)

UL48	E[a]/GD[b]	L (Chambers et al., 1999)	Q6SW84	Cellular Trafficking*	Intracellular capsid transport[?] [†] (largest) Tegument protein [†]	(Mocarski Jr, 2007)
UL48	E[a]/GD[b]	L (Chambers et al., 1999)	Q6SW84	Virion protein	(largest) Tegument protein [†]	(Dunn et al., 2003;Mocarski Jr, 2007)
UL48.5	E[a,b]	N/A	N/A	Virion protein	Located on tips of hexons of capsid [†]	(Baldick and Shenk, 1996;Dunn et al., 2003;Mocarski Jr, 2007)
UL48.5	E[a,b]	N/A	N/A	Cellular Trafficking*	Capsid transport[?] [†] Located on tips of hexons of capsid [†]	(Mocarski Jr, 2007)
UL49	E[a,b]	E/L (Chambers et al., 1999)	Q6SW82	(DNA) Replication	DNA synthesis	(Apweiler et al., 2004;Zhang et al., 2010;Wang et al., 2013a)
UL50	E[a,b]	E /delayed E (Buchkovich et al., 2010)	Q6SW81	Virion protein*	Inner nuclear membrane protein [†]	(Mocarski Jr, 2007)
UL50	E[a,b]	E /delayed E (Buchkovich et al., 2010)	Q6SW81	Latency*	Inner nuclear membrane protein [†]	(Rossetto et al., 2013)
UL50	E[a,b]	E /delayed E (Buchkovich et al., 2010)	Q6SW81	Assembly/Maturation/Egress	Nuclear egress Inner nuclear membrane protein [†]	(Dunn et al., 2003;Mocarski Jr, 2007;Sharma et al., 2014)
UL51	E[a,b]	L (Borst et al., 2013)	F5HGI9	Assembly/Maturation/Egress	DNA cleavage-packaging	(Dunn et al., 2003;Apweiler et al., 2004;Mocarski Jr, 2007;Borst et al., 2013)

UL52	E[a,b]	L (Chambers et al., 1999;Borst et al., 2008)	Q6SW79	Assembly/Maturation/Egress	DNA cleavage-packaging	(Dunn et al., 2003;Borst et al., 2008)
UL52	E[a,b]	L (Chambers et al., 1999;Borst et al., 2008)	Q6SW79	Latency*		(Rossetto et al., 2013)
UL52	E[a,b]	L (Chambers et al., 1999;Borst et al., 2008)	Q6SW79	Cellular Trafficking*	Capsid transport[? †]	(Mocarski Jr, 2007)
UL53	E[a,b]	E (Chambers et al., 1999); L (Dal Monte et al., 2002)	F5HFZ4	Assembly/Maturation/Egress	Nuclear egress<br>Nuclear matrix protein[†]	(Dunn et al., 2003;Mocarski Jr, 2007;Sharma et al., 2014)
UL54	E[a,b]	E (Mocarski Jr, 1996;Chambers et al., 1999)	Q6SW77	(DNA) Replication[($UL54-UL57)]	DNA polymerase[†]<br>Formation of replication compartiment (UL54-UL57)	(Dunn et al., 2003;Apweiler et al., 2004;Mocarski Jr, 2007)
UL55	E[a,b]	E (Mocarski Jr, 1996;Smuda et al., 1997;Chambers et al., 1999)	F5HB53	Entry	Heparan binding[†]<br>Virion glycoprotein(gB)[†]	(Dunn et al., 2003;Apweiler et al., 2004;Mocarski Jr, 2007;Isaacson and Compton, 2009)
UL55	E[a,b]	E (Mocarski Jr, 1996;Smuda et al., 1997;Chambers et al., 1999)	F5HB53	Virion protein	Heparan binding[†]<br>Virion glycoprotein(gB)[†]	(Dunn et al., 2003;Apweiler et al., 2004;Mocarski Jr, 2007;Isaacson and Compton, 2009)

UL55	E[a,b]	E (Mocarski Jr, 1996;Smuda et al., 1997;Chambers et al., 1999)	F5HB53	(DNA) Replication[($UL54-UL57)]	Formation of replication compartiment (UL54-UL57)	(Xiaofei et al., 2012)
UL56	E[a,b]	E (Mocarski Jr, 1996;Chambers et al., 1999)	F5HC79	(DNA) Replication[($UL54-UL57)]	Formation of replication compartiment (UL54-UL57)	(Xiaofei et al., 2012)
UL56	E[a,b]	E (Mocarski Jr, 1996;Chambers et al., 1999)	F5HC79	Assembly/Maturation/Egress	Binds DNA packaging motif[†]<br>DNA cleavage-packaging	(Dunn et al., 2003;Apweiler et al., 2004;Mocarski Jr, 2007;Champier et al., 2008;Borst et al., 2013)
UL56	E[a,b]	E (Mocarski Jr, 1996;Chambers et al., 1999)	F5HC79	Nucleotide repair/modification	Nuclease activity[†]	(Mocarski Jr, 2007;Champier et al., 2008;Borst et al., 2013)
UL57	E[a,b]	E (Mocarski Jr, 1996;Chambers et al., 1999)	F5HDQ6	(DNA) Replication[($UL54-UL57)]	Formation of replication compartiment (UL54-UL57)<br>ssDNA-binding protein[†]	(Dunn et al., 2003;Apweiler et al., 2004;Mocarski Jr, 2007;Woon et al., 2008;Xiaofei et al., 2012)
UL59	D[a]	L (Chambers et al., 1999)	A8T7D9	Unknown		
UL61	E[b]	E (Chambers et al., 1999)	P16818 (AD169)	Latency*		(Goodrum et al., 2002)
UL62	D[a]	N/A	P16819 (AD169)	Unknown		
UL64	D[a]	N/A	P16821 (AD169)	Latency*		(Goodrum et al., 2002)

UL65	MGD[a]/ D[b]	L (Bergallo et al., 2008)	P17148 (AD169)	Latency*		(Goodrum et al., 2002)
UL67	D[a]	L (Chambers et al., 1999)	P16747 (AD169)	Latency*		(Goodrum et al., 2002;Cheung et al., 2006)
UL68	N/A	L ((Chambers et al., 1999)	P16748 (AD169)	Latency*		(Goodrum et al., 2002;Cheung et al., 2006)
UL69	SGD[a]/ GD[b]	E/L (Winkler et al., 1994;Chambers et al., 1999)	Q6SW73	Gene expression/regulation	Binds mRNA cap-binding complex Tegument phosphoprotein	(Dunn et al., 2003;Apweiler et al., 2004;Kalejta, 2008;Kapasi and Spector, 2008;Aoyagi et al., 2010)
UL69	SGD[a]/ GD[b]	E/L (Winkler et al., 1994;Chambers et al., 1999)	Q6SW73	Cellular Trafficking	mRNA export Tegument phosphoprotein	(Kalejta, 2008)
UL69	SGD[a]/ GD[b]	E/L (Winkler et al., 1994;Chambers et al., 1999)	Q6SW73	Modulation of host cell cycle/protein synthesis	G1 arrest Tegument phosphoprotein	(Apweiler et al., 2004;Mocarski Jr, 2007;Kalejta, 2008)
UL69	SGD[a]/ GD[b]	E/L (Winkler et al., 1994;Chambers et al., 1999)	Q6SW73	Virion protein	Tegument phosphoprotein	(Dunn et al., 2003;Mocarski Jr, 2007;Kalejta, 2008)
UL70	E[a,b]	N/A	F5HG51	(DNA) Replication	DNA helicase-primase[†]	(Dunn et al., 2003;Apweiler et al., 2004;Mocarski Jr, 2007;Woon et al., 2008)
UL70	E[a,b]	N/A	F5HG51	Latency*		(Goodrum et al., 2002)

UL71	E[a]/GD[b]	E/L (Schauflinger et al.)	F5HEA3	Assembly/Maturation/Egress	Late envelopment<br>Cytoplasmic egress[?] †<br>Tegument protein †	(Schauflinger et al., 2011;Meissner et al., 2012)
UL71	E[a]/GD[b]	E/L (Schauflinger et al.)	F5HEA3	Virion protein	Tegument protein †	(Apweiler et al., 2004;Mocarski Jr, 2007)
UL72	MGD[a]/ GD[b]	E/L (Chambers et al., 1999)	Q6SW70	Virion protein		(Mocarski Jr, 2007)
UL72	MGD[a]/ GD[b]	E/L (Chambers et al., 1999)	Q6SW70	Nucleotide repair/modification	Deoxyuridine triphosphatase homologue (not active) †	(Dunn et al., 2003;Apweiler et al., 2004;Mocarski Jr, 2007)
UL73	E[a,b]	E/L (Chambers et al., 1999)	F5HHQ0	Entry	Virion glycoprotein (gN) †	(Dunn et al., 2003;Mocarski Jr, 2007)
UL73	E[a,b]	E/L (Chambers et al., 1999)	F5HHQ0	Virion Protein	Virion glycoprotein (gN) †	(Apweiler et al., 2004;Mocarski Jr, 2007)
UL73	E[a,b]	E/L (Chambers et al., 1999)	F5HHQ0	Latency*		(Goodrum et al., 2002)
UL74	MGD[a]/ GD[b]	L (Huber and Compton, 1998)	F5HGP1	Viral spread	Cell-to-cell spread<br>Virion glycoprotein (gO) †	(Kinzler and Compton, 2005)
UL74	MGD[a]/ GD[b]	L (Huber and Compton, 1998)	F5HGP1	Assembly/Maturation/Egress	Secondary Envelopment<br>Virion glycoprotein (gO) †	(Jiang et al., 2008)
UL74	MGD[a]/ GD[b]	L (Huber and Compton, 1998)	F5HGP1	Entry	Virion glycoprotein (gO) †	(Mocarski Jr, 2007)

UL74	MGD[a]/ GD[b]	L (Huber and Compton, 1998)	F5HGP1	Immunomodulation	Reduces the inhibitory effect of gH- and gB-specific antibodies Virion glycoprotein (gO)†	(Jiang et al., 2011)
UL74	MGD[a]/ GD[b]	L (Huber and Compton, 1998)	F5HGP1	Virion protein	Virion glycoprotein (gO)†	(Apweiler et al., 2004;Mocarski Jr, 2007)
UL75	E[a,b]	E/L (Chambers et al., 1999); L (Mocarski Jr, 1996)	Q6SW67	Virion protein	Virion glycoprotein (gH)†	(Mocarski Jr, 2007)
UL75	E[a,b]	E/L (Chambers et al., 1999); L (Mocarski Jr, 1996)	Q6SW67	Entry	Virion glycoprotein (gH)†	(Apweiler et al., 2004;Mocarski Jr, 2007)
UL75	E[a,b]	E/L (Chambers et al., 1999); L (Mocarski Jr, 1996)	Q6SW67	Latency*	Virion glycoprotein (gH)†	(Goodrum et al., 2002)
UL76	E[a]/GD[b]	L (Wang et al., 2004)	Q6SW66	Virion protein	Virion-associated regulatory protein†	(Mocarski Jr, 2007)
UL76	E[a]/GD[b]	L (Wang et al., 2004)	Q6SW66	Apoptosis	Inducer of DNA damage Virion-associated regulatory protein†	(Siew et al., 2009;Costa et al., 2013)
UL76	E[a]/GD[b]	L (Wang et al., 2004)	Q6SW66	Gene expression/regulation	Regulator of UL77 expression Virion-associated regulatory protein†	(Isomura et al., 2010)

UL76	E[a]/GD[b]	L (Wang et al., 2004)	Q6SW66	(DNA) Replication	Negative regulator of replication<br>Virion-associated regulatory protein †	(Wang et al., 2004)
UL76	E[a]/GD[b]	L (Wang et al., 2004)	Q6SW66	Latency*	Virion-associated regulatory protein †	(Goodrum et al., 2002;Wang et al., 2004)
UL77	E[a,b]	E (Chambers et al., 1999)	Q6SW65	Assembly/Maturation/Egress	DNA packaging<br>Portal capping protein †	(Dunn et al., 2003;Apweiler et al., 2004;Mocarski Jr, 2007;Meissner et al., 2011)
UL78	D[a,b]	E (Chambers et al., 1999)	F5HET1	Immunomodulation	Modulation chemo- and/or cytokine receptors<br>Chemokine receptor-like protein<br>GPCR family †	(Beisser et al., 2002;Mocarski Jr, 2007;Tadagaki et al., 2012)
UL78	D[a,b]	E (Chambers et al., 1999)	F5HET1	Cell tropism/Cell type-specific replication	Involved in cell tropism epithelial cells<br>Chemokine receptor-like protein<br>GPCR family †	(O'Connor and Shenk, 2012)
UL79	E[a,b]	E (Isomura et al., 2011)	Q6SW63	Gene expression/regulation	Required for accumulation late transcripts	(Isomura et al., 2011;Perng et al., 2011)
UL79	E[a,b]	E (Isomura et al., 2011)	Q6SW63	Latency*		(Rossetto et al., 2013)
UL80	E[a,b]	L (Welch et al., 1991)	Q6SW62	Latency*	Protease and capsid assembly protein †	(Goodrum et al., 2002)
UL80	E[a,b]	L (Welch et al., 1991)	Q6SW62	Assembly/Maturation/Egress	Protease and capsid assembly protein †	(Dunn et al., 2003;Mocarski Jr, 2007)

UL80	E[a,b]	L (Welch et al., 1991)	Q6SW62	(DNA) Replication*	Involvement nuclear localization signals<br>Protease and capsid assembly protein †	(Nguyen et al., 2008)
UL80.5	E[b]	N/A	F5HE40	Assembly/Maturation/Egress	Early capsid formation	(Loveland et al., 2007;Mocarski Jr, 2007)
UL81ast/LUNA	N/A	L (Keyes et al., 2012)	N/A	Latency	Established LAT	(Goodrum et al., 2002;Bego et al., 2005;Bego et al., 2011;Rossetto et al., 2013)
UL82/pp71	SGD[a]/ GD[b]	L (Mocarski Jr, 1996;Chambers et al., 1999)	F5HBC6	Virion protein	Tegument phosphoprotein †<br>UL82 family †	(Dunn et al., 2003;Mocarski Jr, 2007)
UL82/pp71	SGD[a]/ GD[b]	L (Mocarski Jr, 1996;Chambers et al., 1999)	F5HBC6	Gene expression/regulation	Activator of MIEP<br>Tegument phosphoprotein †<br>UL82 family †	(Dunn et al., 2003;Mocarski Jr, 2007;Penkert and Kalejta, 2012)
UL82/pp71	SGD[a]/ GD[b]	L (Mocarski Jr, 1996;Chambers et al., 1999)	F5HBC6	Modulation of host cell cycle/protein synthesis	Interaction with Rb family<br>Tegument phosphoprotein †<br>UL82 family †	(Apweiler et al., 2004;Penkert and Kalejta, 2012)
UL82/pp71	SGD[a]/ GD[b]	L (Mocarski Jr, 1996;Chambers et al., 1999)	F5HBC6	Immunomodulation	Granzyme M degradation<br>Tegument phosphoprotein †<br>UL82 family †	(Penkert and Kalejta, 2012)

UL82/pp71	SGD[a]/ GD[b]	L (Mocarski Jr, 1996;Chambers et al., 1999)	F5HBC6	Latency*	Prevents DAXX degradation Tegument phosphoprotein † UL82 family †	(Penkert and Kalejta, 2012)
UL83/pp65	D[a,b]	L (Chambers et al., 1999); E/L (Mocarski Jr, 1996)	Q6SW59	Cell tropism/Cell type-specific replication*	May be involved in cell tropism epithelial cells	(Womack, 2011)
UL83/pp65	D[a,b]	L (Chambers et al., 1999); E/L (Mocarski Jr, 1996)	Q6SW59	Virion protein	Major Tegument phosphoprotein † UL82 family †	(Dunn et al., 2003;Mocarski Jr, 2007)
UL83/pp65	D[a,b]	L (Chambers et al., 1999); E/L (Mocarski Jr, 1996)	Q6SW59	Gene expression/regulation	Activator of MIEP Major Tegument phosphoprotein † UL82 family †	(Cristea et al., 2010;Arcangeletti et al., 2011)
UL83/pp65	D[a,b]	L (Chambers et al., 1999); E/L (Mocarski Jr, 1996)	Q6SW59	Immunomodulation	Suppression IFN response Major Tegument phosphoprotein † UL82 family †	(Apweiler et al., 2004;Mocarski Jr, 2007)
UL84	E[a,b]	E/L (Mocarski Jr, 1996;Chambers et al., 1999)	F5HB40	(DNA) Replication	Required for oriLyt-dependent DNA replication(through protein-protein interaction)	(Dunn et al., 2003;Colletti et al., 2007;Mocarski Jr, 2007;Woon et al., 2008;Gao and Pari, 2009;Kagele et al., 2009;Strang et al., 2012)

UL84	E[a,b]	E/L (Mocarski Jr, 1996;Chambers et al., 1999)	F5HB40	Viral Growth	Nucleoplasmic shuttling essential for viral growth	(Gao et al., 2010)
UL84	E[a,b]	E/L (Mocarski Jr, 1996;Chambers et al., 1999)	F5HB40	Latency*		(Goodrum et al., 2002;Rossetto et al., 2013)
UL85	E[a,b]	E/L (Chambers et al., 1999)	F5HIN9	Assembly/Maturation/Egress	Minor capsid protein †	(Dunn et al., 2003;Mocarski Jr, 2007)
UL86	E[a,b]	E/L (Chambers et al., 1999); L (Mocarski Jr, 1996)	F5HGT1	Assembly/Maturation/Egress	Major capsid protein †	(Dunn et al., 2003;Mocarski Jr, 2007)
UL87	E[a,b]	E (Isomura et al., 2010)	Q6SW55	Gene expression/regulation	Required for accumulation late transcripts	(Isomura et al., 2011)
UL87	E[a,b]	E (Isomura et al., 2011)	Q6SW55	Latency*		(Goodrum et al., 2002;Rossetto et al., 2013)
UL88	MGD[a]/ D[b]	N/A	F5H9F9	Virion protein	Tegument protein †	(Dunn et al., 2003;Apweiler et al., 2004;Mocarski Jr, 2007)
UL88	MGD[a]/ D[b]	N/A	F5H9F9	Assembly/Maturation/Egress*	Cytoplasmic egress † Tegument protein †	(Mocarski Jr, 2007)
UL89	E[a,b]	E/L (Chambers et al., 1999)	F5HCU8	Viral growth	shRNA against UL89 inhibits of formation of replicative infectious particles	(Thoma and Bogner, 2010)
UL89.1	E[a,b]	N/A	F5HCU8	Assembly/Maturation/Egress	Terminase ATPase subunit †	(Dunn et al., 2003;Apweiler et al., 2004;Mocarski Jr, 2007)

UL89.2	D[a]	N/A	F5HCU8	Assembly/Maturation/Egress	Terminase ATPase subunit †	(Dunn et al., 2003;Apweiler et al., 2004;Mocarski Jr, 2007)
UL90	E[a]	N/A	P16796	Unknown		
UL91	E[a,b]	L (Chambers et al., 1999)	F5HFJ8	Gene expression/regulation	Essential for transcription of viral true late (γ2) genes	(Omoto and Mocarski, 2013)
UL92	E[a,b]	L (Chambers et al., 1999)	F5HAS7	Gene expression/regulation	Essential for transcription of viral true late (γ2) genes	(Omoto and Mocarski, 2014)
UL93	E[a,b]	L (Chambers et al., 1999)	Q6SW50	Assembly/Maturation/Egress*	Possible role in DNA packaging<br>Tegument protein †	(Apweiler et al., 2004)
UL93	E[a,b]	L (Chambers et al., 1999)	Q6SW50	Virion protein	Tegument protein †	(Mocarski Jr, 2007)
UL93	E[a,b]	L (Chambers et al., 1999)	Q6SW50	Cellular trafficking*	Capsid transport? †<br>Tegument protein †	(Mocarski Jr, 2007)
UL94	E[a]/GD[b]	L (Wing et al., 1996;Chambers et al., 1999)	F5HAC7	Virion protein	Tegument protein †	(Dunn et al., 2003;Apweiler et al., 2004;Mocarski Jr, 2007;Phillips et al., 2012)
UL94	E[a]/GD[b]	L (Wing et al., 1996;Chambers et al., 1999)	F5HAC7	Assembly/Maturation/Egress	Secondary envelopment<br>Cytoplasmic egress? †<br>Tegument protein †	(Mocarski Jr, 2007;Phillips and Bresnahan, 2012)
UL95	E[a,b]	E/L (Wing and Huang, 1995)	Q6SW48	Gene expression/regulation	Required for accumulation late transcripts	(Isomura et al., 2011)
UL95	E[a,b]	E/L (Wing and Huang, 1995)	Q6SW48	Assembly/Maturation/Egress*	Encapsidation chaperone protein? †	(Mocarski Jr, 2007)
UL95	E[a,b]	E/L (Wing and Huang, 1995)	Q6SW48	Latency*		(Rossetto et al., 2013)

UL96	E[a]/GD[b]	E/L (Chambers et al., 1999)	F5H8R6	Virion protein	Tegument protein †	(Apweiler et al., 2004;Mocarski Jr, 2007;Tandon and Mocarski, 2011)
UL96	E[a]/GD[b]	E/L (Chambers et al., 1999)	F5H8R6	Assembly/Maturation/Egress	Stabilizes pp150-associated nucleocapsids Tegument protein †	(Tandon and Mocarski, 2011)
UL97	MGD[a]/ GD[b]	E/L (Chambers et al., 1999)	Q6SW46	Modulation of host cell cycle/protein synthesis	Mimics cdc2/CDK1 † Tegument protein †	(Apweiler et al., 2004;Mocarski Jr, 2007)
UL97	MGD[a]/ GD[b]	E/L (Chambers et al., 1999)	Q6SW46	Gene expression/regulation	Regulator of MIEP Tegument protein †	(Bigley et al., 2013)
UL97	MGD[a]/ GD[b]	E/L (Chambers et al., 1999)	Q6SW46	Virion protein	Tegument protein †	(Mocarski Jr, 2007)
UL97	MGD[a]/ GD[b]	E/L (Chambers et al., 1999)	Q6SW46	(DNA) Replication	Tegument protein †	(Mocarski Jr, 2007)
UL97	MGD[a]/ GD[b]	E/L (Chambers et al., 1999)	Q6SW46	Assembly/Maturation/Egress	DNA packaging Nuclear egress Tegument protein †	(Mocarski Jr, 2007)
UL98	E[a,b]	E/L (Chambers et al., 1999)	F5HF49	Nucleotide repair/modification	Deoxyribonuclease †	(Dunn et al., 2003;Apweiler et al., 2004;Mocarski Jr, 2007)
UL98	E[a,b]	E/L (Chambers et al., 1999)	F5HF49	Latency*		(Goodrum et al., 2002)
UL99/pp28	E[a,b]	L (Mocarski Jr, 1996)	F5HI87	Virion protein	Myristylated tegument protein †	(Dunn et al., 2003;Apweiler et al., 2004;Mocarski Jr, 2007)
UL99/pp28	E[a,b]	L (Mocarski Jr, 1996)	F5HI87	Latency*	Myristylated tegument protein †	(Goodrum et al., 2002)

UL99/pp28	E[a,b]	L (Mocarski Jr, 1996)	F5HI87	Assembly/Maturation/Egress	Secondary envelopment Cytoplasmic egress † Myristylated tegument protein †	(Silva et al., 2003;Mocarski Jr, 2007)
UL99/pp28	E[a,b]	L (Mocarski Jr, 1996)	F5HI87	(DNA) Replication	Interaction with UL94 essential for replication	(Phillips et al., 2012)
UL100	E[a,b]	E/L (Chambers et al., 1999); L (Mocarski Jr, 1996)	Q6SW43	Entry	Virion glycoprotein (gM) †	(Dunn et al., 2003;Mocarski Jr, 2007)
UL100	E[a,b]	E/L (Chambers et al., 1999); L (Mocarski Jr, 1996)	Q6SW43	Assembly/Maturation/Egress	gM trafficking in virion assembly Virion glycoprotein (gM) †	(Krzyzaniak et al., 2007)
UL100	E[a,b]	E/L (Chambers et al., 1999); L (Mocarski Jr, 1996)	Q6SW43	(DNA) Replication	Deletion of cytoplasmic tail leads to replication defective virus Virion glycoprotein (gM) †	(Krzyzaniak et al., 2007)
UL100	E[a,b]	E/L (Chambers et al., 1999); L (Mocarski Jr, 1996)	Q6SW43	Virion protein	Virion glycoprotein (gM) †	(Dunn et al., 2003;Mocarski Jr, 2007)
UL102	E[a,b]	L (Chambers et al., 1999); IE/E (Smith and Pari, 1995b)	F5HIG1	(DNA) Replication	Component of DNA helicase-primase †	(Smith and Pari, 1995b;Dunn et al., 2003;Apweiler et al., 2004;Mocarski Jr, 2007)
UL103	MGD[a]/ GD[b]	L (Chambers et al., 1999)	F5HA10	Virion protein	Tegument protein †	(Apweiler et al., 2004;Mocarski Jr, 2007)
UL103	MGD[a]/ GD[b]	L (Chambers et al., 1999)	F5HA10	Assembly/Maturation/Egress	Regulates particle egress	(Mocarski Jr, 2007;Ahlqvist and Mocarski, 2011)

UL104	E[a,b]	E (Chambers et al., 1999)	F5HBR4	Assembly/Maturation/Egress	DNA encapsidation †	(Dunn et al., 2003;Apweiler et al., 2004;Mocarski Jr, 2007)
UL105	E[a,b]	L (Chambers et al., 1999)	F5HEN8	(DNA) Replication	Component of DNA helicase-primase †	(Dunn et al., 2003;Apweiler et al., 2004;Mocarski Jr, 2007)
UL105	E[a,b]	L (Chambers et al., 1999)	F5HEN8	Latency*		(Goodrum et al., 2002)
UL108	MGD[a]	L (Chambers et al., 1999)	P16829 (AD169)	Latency*		(Goodrum et al., 2002;Cheung et al., 2006)
UL109	D[a]	L (Chambers et al., 1999)	P16738 (AD169)	Cell tropism/cell type-specific replication*	May be involved in cell tropism epithelial cells	(Womack, 2011)
UL110	D[a]	IE/E/L (Chambers et al., 1999)	P16830 (AD169)	Cell tropism/cell type-specific replication*	May be involved in cell tropism epithelial cells	(Womack, 2011)
UL110	D[a]	IE/E/L (Chambers et al., 1999)	P16830 (AD169)	Latency*		(Goodrum et al., 2002;Cheung et al., 2006)
UL111A	D[a]	E/L (Chambers et al., 1999); L (Chang et al., 2004)	F5HC71	Cell tropism/cell type-specific replication*	May be involved in cell tropism epithelial cells	(Womack, 2011)
UL111A	D[a]	E/L (Chambers et al., 1999); L (Chang et al., 2004)	F5HC71	Immunomodulation	vIL10	(Dunn et al., 2003;Apweiler et al., 2004;Mocarski Jr, 2007;Jenkins et al., 2008;Cheung et al., 2009;Avdic et al., 2011;Avdic et al., 2013)

UL111A	D[a]	E/L (Chambers et al., 1999); L (Chang et al., 2004)	F5HC71	Latency	Established LAT	(Cheung et al., 2006;Avdic et al., 2011;Rossetto et al., 2013)
UL112	SGD[a]/ GD[b]	E (Mocarski Jr, 1996;Chambers et al., 1999)	Q6SW37	Gene expression/regulation	Transcriptional activator †	(Mocarski Jr, 2007)
UL112	SGD[a]/ GD[b]	E (Mocarski Jr, 1996;Chambers et al., 1999)	Q6SW37	(DNA) Replication	Organisation of DNA replication †	(Kim and Ahn, 2010)
UL113	SGD[a]/ GD[b]	E/L (Chambers et al., 1999); L (Mocarski Jr, 1996)	Q6SW37	Gene expression/regulation	Transcriptional activator †	(Mocarski Jr, 2007)
UL113	SGD[a]/ GD[b]	E/L (Chambers et al., 1999); L (Mocarski Jr, 1996)	Q6SW37	(DNA) Replication	Promotes DNA replication	(Mocarski Jr, 2007;Kim and Ahn, 2010)
UL114	MGD[a]/ GD[b]	E (Chambers et al., 1999)	*F5HI85*	Nucleotide repair/modification	Uracyl-DNA glycosylase †	(Dunn et al., 2003;Apweiler et al., 2004;Mocarski Jr, 2007)
UL114	MGD[a]/ GD[b]	E (Chambers et al., 1999)	F5HI85	(DNA) Replication	Increases efficiency of DNA replication Interaction with UL54 Temporal regulation of DNA replication †	(Prichard et al., 1996;Mocarski Jr, 2007;Ranneberg-Nilsen et al., 2008;Strang and Coen, 2010)
UL114	MGD[a]/ GD[b]	E (Chambers et al., 1999)	F5HI85	Latency*		(Rossetto et al., 2013)
UL115	E[a,b]	L (Mocarski Jr, 1996)	F5HCH8	Virion protein	Virion glycoprotein (gL) †	(Dunn et al., 2003;Apweiler et al., 2004;Mocarski Jr, 2007)

UL115	E[a,b]	L (Mocarski Jr, 1996)	F5HCH8	Entry	Virion glycoprotein (gL) †	(Dunn et al., 2003;Apweiler et al., 2004;Mocarski Jr, 2007)
UL115	E[a,b]	L (Mocarski Jr, 1996)	F5HCH8	Latency*		(Cheung et al., 2006)
UL116	D[a,b]	E/L (Chambers et al., 1999); L (Mocarski Jr, 1996)	Q6SW34	Unknown	Putative membrane glycoprotein †	
UL117	SGD[a]/ GD[b]	L (Mocarski Jr, 1996)	F5HFA5	(DNA) replication	Involved in maturation of viral replication compartments	(Qian et al., 2008)
UL118	D[b]	E (Chambers et al., 1999); L (Mocarski Jr, 1996)	F5HC14	Immunomodulation($UL118-UL119)	Viral Fc Gamma receptor	(Atalay et al., 2002;Apweiler et al., 2004)
UL119	D[a,b]	E (Chambers et al., 1999); L (Mocarski Jr, 1996)	F5HC14	Immunomodulation($UL118-UL119)	Viral Fc Gamma receptor	(Dunn et al., 2003;Apweiler et al., 2004;Mocarski Jr, 2007)
UL120	D[b]	L (Chambers et al., 1999)	Q6SW31	Unknown	Putative membrane glycoprotein †<br>UL120 family †	
UL121	D[a,b]	L (Chambers et al., 1999)	F5HD27	Unknown	Putative membrane glycoprotein †<br>UL120 family †	
UL122	E[a,b]	IE/E (Mocarski Jr, 1996;Chambers et al., 1999)	Q6SW29	Gene expression/regulation	Negative regulator MIEP<br>IE2	(Dunn et al., 2003;Apweiler et al., 2004;Mocarski Jr, 2007;Martinez et al., 2014)

UL122	E[a,b]	IE/E (Mocarski Jr, 1996;Chambers et al., 1999)	Q6SW29	Latency*	IE2	(Goodrum et al., 2002)
UL122	E[a,b]	IE/E (Mocarski Jr, 1996;Chambers et al., 1999)	Q6SW29	Modulation of host cell cycle/protein synthesis	Repression p53<br>IE2	(Apweiler et al., 2004;Kwon et al., 2012)
UL123	SGD[a]/ GD[b]	IE (Mocarski Jr, 1996;Chambers et al., 1999)	F5HCM1	Gene expression/regulation	Antagonizes histone acetylation and affects nucleosome organization to activate transcription<br>IE1	(Apweiler et al., 2004;Nevels et al., 2004;Mocarski Jr, 2007;Zalckvar et al., 2013)
UL123	SGD[a]/ GD[b]	IE (Mocarski Jr, 1996;Chambers et al., 1999)	F5HCM1	Modulation of host cell cycle/protein synthesis	IE1	(Apweiler et al., 2004)
UL123	SGD[a]/ GD[b]	IE (Mocarski Jr, 1996;Chambers et al., 1999)	F5HCM1	Immunomodulation	Inhibition of type I IFN signaling via STAT2<br>Disruption of IL6 signaling via STAT3<br>Elicits a type II IFN-like response via STAT1<br>IE1	(Apweiler et al., 2004;Paulus et al., 2006;Knoblach et al., 2011;Reitsma et al., 2013)
UL123	SGD[a]/ GD[b]	IE (Mocarski Jr, 1996;Chambers et al., 1999)	F5HCM1	Latency*	Binds nucleosomes, role in latency suggested<br>IE1	(Rossetto et al., 2013;Mucke et al., 2014)
UL124	SGD[a]/ D[b]	E (Chambers et al., 1999)	F5HHS3	Latency*	Membrane glycoprotein<br>†	(Goodrum et al., 2002)
UL125	N/A	E (Chambers et al., 1999)	P16835 (AD169)	Latency*		(Goodrum et al., 2002)

UL126A	N/A	N/A	N/A	Latency*		(Rossetto et al., 2013)
UL127	D[a]	E (Lashmit et al., 2004)	P16771 (AD169)	Unknown		
UL128	D[b]	E (Chambers et al., 1999)	P16837 (AD169)	Latency*	Putative secreted protein †	(Goodrum et al., 2002)
UL128	D[b]	E (Chambers et al., 1999)	P16837 (AD169)	Cell tropism/Cell type-specific replication	Involved in cell tropism endothelial cells Putative secreted protein †	(Mocarski Jr, 2007;Ryckman et al., 2008;Stanton et al., 2010)
UL128	D[b]	E (Chambers et al., 1999)	P16837 (AD169)	Immunomodulation	Modulation chemo- and/or cytokines Modulation of monocyte migration Putative secreted protein †	(Mocarski Jr, 2007;Straschewski et al., 2011;Zheng et al., 2012)
UL129	MGD[a]	L (Chambers et al., 1999)	P16838 (AD169)	Unknown		
UL130	D[a,b]	E/L (Chambers et al., 1999)	F5HCP3	Cell tropism/Cell type-specific replication	Involved in cell tropism endothelial and epithelial cells Putative secreted protein †	(Patrone et al., 2005;Ryckman et al., 2008;Stanton et al., 2010)
UL131A	N/A	L (Akter et al., 2003)	F5HET4	Virion protein	Putative secreted protein †	(Apweiler et al., 2004)
UL131A	N/A	L (Akter et al., 2003)	F5HET4	Cell tropism/Cell type-specific replication	Involved in cell tropism endothelial and epithelial cells Putative secreted protein †	(Schuessler et al., 2008;Stanton et al., 2010;Schuessler et al., 2012)
UL132	MGD[a]/ D[b]	E/L (Chambers et al., 1999)	F5HGU6	Cell tropism/Cell type-specific replication*	May be involved in cell tropism epithelial cells Virion glycoprotein †	(Womack, 2011)

UL132	MGD[a]/ D[b]	E/L (Chambers et al., 1999)	F5HGU6	Virion protein	Virion glycoprotein †	(Spaderna et al., 2005;Mocarski Jr, 2007)
UL132	MGD[a]/ D[b]	E/L (Chambers et al., 1999)	F5HGU6	Latency*	Virion glycoprotein †	(Goodrum et al., 2002)
UL133	N/A	L ? (Bughio et al., 2013)	Q6SW10	Assembly/Maturation/Egress($UL 133-UL138)	Tegumentation and secondary envelopment Putative membrane protein †	(Bughio et al., 2013)
UL133	N/A	L ? (Bughio et al., 2013)	Q6SW10	Latency*	Putative membrane protein †	(Goodrum et al., 2002;Petrucelli et al., 2009;Rossetto et al., 2013)
UL134	N/A	L ? (Bughio et al., 2013)	Q66M59 (**)	Assembly/Maturation/Egress($UL 133-UL138)	Tegumentation and secondary envelopment	(Bughio et al., 2013)
UL135	N/A	L ? (Bughio et al., 2013)	F5HAQ7	Latency*	Putative secreted protein †	(Goodrum et al., 2002;Rossetto et al., 2013)
UL135	N/A	L ? (Bughio et al., 2013)		Assembly/Maturation/Egress($UL 133-UL138)	Tegumentation and secondary envelopment Putative secreted protein †	(Bughio et al., 2013)
UL136	N/A	L ? (Bughio et al., 2013)	F5HF35	Assembly/Maturation/Egress($UL 133-UL138)	Tegumentation and secondary envelopment Putative membrane protein †	(Bughio et al., 2013)
UL138	N/A	E/L (Petrucelli et al., 2009); L? (Bughio et al., 2013)	F5HGQ8	Assembly/Maturation/Egress($UL 133-UL138)	Tegumentation and secondary envelopment Putative membrane protein †	(Bughio et al., 2013)

UL138	N/A	E/L (Petrucelli et al., 2009); L? (Bughio et al., 2013)	F5HGQ8	Latency	Established LAT<br>Putative membrane protein [†]	(Goodrum et al., 2002;Goodrum et al., 2007;Petrucelli et al., 2009;Rossetto et al., 2013)
UL138	N/A	E/L (Petrucelli et al., 2009); L? (Bughio et al., 2013)	F5HGQ8	Immunomodulation	Modulation of TNF signaling<br>Putative membrane protein [†]	(Le et al., 2011;Montag et al., 2011)
UL139	N/A	E/L (Bradley et al., 2008)	Q6SW14	Immunomodulation*	Homology with CD24<br>Putative membrane glycoprotein [†]	(Qi et al., 2006)
UL140	N/A	L (Ma et al., 2013)	F5HCK7	Unknown	Putative membrane protein [†]	
UL141	N/A	L (Ma et al., 2013)	Q6RJQ3	Immunomodulation	Modulation of NK cell signaling/function (via TRAIL)<br>Membrane glycoprotein [†]<br>UL14 family [†]	(Apweiler et al., 2004;Mocarski Jr, 2007;Prod'homme et al., 2010;Smith et al., 2013)
UL142	N/A	L (Wills et al., 2005)	F5HHH2	Immunomodulation	MHC-I homologue<br>Putative membrane glycoprotein<br>UL18 family [†]	(Wills et al., 2005;Mocarski Jr, 2007;Ashiru et al., 2009)
UL144	N/A	E (Poole et al., 2008)	F5HAM0	Immunomodulation	Modulation of chemo- and/or cytokines	(Poole et al., 2006;Poole et al., 2008)
UL144	N/A	E (Poole et al., 2008)	F5HAM0	Latency	Established LAT	(Poole et al., 2013)
UL145	N/A	L (Wang et al., 2011); IE (Raftery et al., 2009)	F5HF44	Latency*		(Goodrum et al., 2002)

UL146	D[a]	E/L (Chambers et al., 1999)(annotated as UL152)	Q6SWX2 (**)	Immunomodulation	vCXCL1 (CXCR1 and CXCR2)<br>Secreted glycoprotein †<br>UL146 family †	(Dunn et al., 2003;Apweiler et al., 2004;Mocarski Jr, 2007;Luttichau, 2010)
UL147	D[a]	E/L (Chambers et al., 1999)	F5HA06	Immunomodulation*	vCXCL2<br>Putative secreted glycoprotein †<br>UL146 family †	(Dunn et al., 2003;Apweiler et al., 2004;Mocarski Jr, 2007;Heo et al., 2008;Luttichau, 2010)
UL147A	N/A	E/L (Lurain et al., 2006)	F5H8R0	Unknown	Putative membrane protein †	
UL148	N/A	E/L (Lurain et al., 2006)	F5H8Q3	Cell tropism/Cell type-specific replication*	May be involved in cell tropism epithelial cells<br>Putative membrane glycoprotein †	(Womack, 2011)
UL148A	N/A	N/A	F5HE74	Unknown	Putative membrane protein †	
UL148B	N/A	N/A	F5HAK6	Unknown	Putative membrane protein †	
UL148C	N/A	N/A	F5HDE7	Unknown	Putative membrane protein †	
UL148D	N/A	N/A	F5HHL7	Unknown	Putative membrane protein †	
UL149	N/A	N/A	Q6PMP2 (**)	Unknown		
UL150	N/A	N/A	Q6SW05	Latency*	Putative secreted protein †	(Goodrum et al., 2002)
US1	D[a,b]	IE (Mocarski Jr, 1996)	Q6SW03	Unknown	US1 family †	

US2	D[a,b]	IE (Hesse et al., 2013); E (Mocarski Jr, 1996)	F5HE05	Immunomodulation	Degradation of MHC-I<br>Membrane glycoprotein †<br>US2 family †	(Oresic et al., 2006;Oresic and Tortorella, 2008;Noriega et al., 2012;Hesse et al., 2013)
US3	D[a,b]	IE (Mocarski Jr, 1996;Chambers et al., 1999)	F5HEU0	Immunomodulation	Degradation of MHC-I<br>Membrane glycoprotein †<br>US2 family †	(Noriega et al., 2012)
US6	D[a,b]	E/L (Jones et al., 1991;Chambers et al., 1999)	Q6SW00	Immunomodulation	Modulation of T- and NK-cell signaling/function<br>Putative membrane glycoprotein †<br>US6 family †	(Dunn et al., 2003;Apweiler et al., 2004;Mocarski Jr, 2007;Dugan and Hewitt, 2008;Kim et al., 2008)
US7	D[a,b]	E/L (Jones et al., 1991;Chambers et al., 1999)	F5HDD3	Immunomodulation*	US7 is modulated by miRNA<br>Membrane glycoprotein †<br>US6 family †	(Apweiler et al., 2004;Tirabassi et al., 2011)
US8	D[a,b]	E (Jones et al., 1991;Chambers et al., 1999)	F5HB52	Immunomodulation	Binds MHC-I †<br>Membrane glycoprotein †<br>US6 family †	(Dunn et al., 2003;Apweiler et al., 2004;Mocarski Jr, 2007)
US9	D[a,b]	E (Jones et al., 1991;Chambers et al., 1999)	F5HC33	Immunomodulation*	Membrane glycoprotein †<br>US6 family †	(Apweiler et al., 2004)

US9	D[a,b]	E (Jones et al., 1991;Chambers et al., 1999)	F5HC33	Viral spread	Specifically cell-to-cell transmission in endothelial cells Membrane glycoprotein † US6 family †	(Pereira et al., 1995;Mocarski Jr, 2007)
US10	D[a,b]	E (Jones et al., 1991;Chambers et al., 1999)	F5HFJ7	Immunomodulation	Delays MHC-I trafficking † Membrane glycoprotein † US6 family †	(Dunn et al., 2003;Apweiler et al., 2004;Mocarski Jr, 2007)
US11	D[a,b]	E (Jones et al., 1991;Chambers et al., 1999)	Q6SVZ5	Immunomodulation	Degradation MHC-I † Membrane glycoprotein † US6 family †	(Wiertz et al., 1996;Dunn et al., 2003;Apweiler et al., 2004;Mocarski Jr, 2007;Hesse et al., 2013)
US12	D[a,b]	E (Chambers et al., 1999)	F5HE44	Unknown	Putative multiple transmembrane protein † US12 family †	
US13	MGD[a]/ D[b]	E (Chambers et al., 1999)	F5H9I4	Unknown	Putative multiple transmembrane protein † US12 family †	
US14	D[a,b]	E (Chambers et al., 1999)	F5HD92	Unknown	Putative multiple transmembrane protein † US12 family †	

US15	D[a,b]	E/L (Chambers et al., 1999)	F5HFH0	Unknown	Putative multiple transmembrane protein † US12 family †	
US16	D[a,b]	E (Chambers et al., 1999)	Q6SVZ0	Cell tropism/Cell type-specific replication	Involved in cell tropism epithelial and endothelial cells Putative multiple transmembrane protein † US12 family †	(Mocarski Jr, 2007;Bronzini et al., 2012)
US17	D[a,b]	E (Chambers et al., 1999)	F5H9N9	Assembly/Maturation/Egress	Final virion assembly and egress Putative multiple transmembrane protein † US12 family †	(Gurczynski et al., 2013)
US17	D[a,b]	E (Chambers et al., 1999)	F5H9N9	Immunomodulation	Controls virion composition to elicit a balanced immune response	(Gurczynski et al., 2013)
US17	D[a,b]	E (Chambers et al., 1999)	F5H9N9	Latency*		(Rossetto et al., 2013)
US18	D[a,b]	E (Chambers et al., 1999); L (Guo and Huang, 1993)	F5HE69	Unknown	Putative multiple transmembrane protein † US12 family †	
US19	D[a,b]	E (Guo and Huang, 1993;Chambers et al., 1999)	F5HAR3	Viral growth	Temperance in epithelial cells Putative multiple transmembrane protein † US12 family †	(Mocarski Jr, 2007)

US20	D[a,b]	E (Guo and Huang, 1993;Chambers et al., 1999)	F5HGH8	Unknown	Putative multiple transmembrane protein † US12 family †	
US21	D[a,b]	N/A	F5HHT6	Unknown	Putative multiple transmembrane protein † US12 family †	
US22	D[a,b]	E (Guo and Huang, 1993;Chambers et al., 1999)	F5HDC7	Cell tropism/Cell type-specific replication*	May be involved in cell tropism epithelial cells Tegument protein † US22 family †	(Womack, 2011)
US22	D[a,b]	E (Guo and Huang, 1993;Chambers et al., 1999)	F5HDC7	Virion protein	Tegument protein † US22 family †	(Apweiler et al., 2004;Mocarski Jr, 2007)
US23	MGD[a]/ GD[b]	E (Chambers et al., 1999)	F5HAZ3	Virion protein	Tegument protein † US22 family †	(Apweiler et al., 2004;Mocarski Jr, 2007)
US24	D[a]/GD[b]	E (Chambers et al., 1999)	F5H8S6	Virion protein	Tegument protein † US22 family †	(Apweiler et al., 2004;Mocarski Jr, 2007)
US24	D[a]/GD[b]	E (Chambers et al., 1999)	F5H8S6	Gene expression/regulation	Important for IE, E and L gene expression Tegument protein † US22 family †	(Feng et al., 2006)
US25	D[a,b]	E/L (Chambers et al., 1999)	P17145 (AD169)	Unknown		
US26	SGD[a]/ GD[b]	E (Chambers et al., 1999)	F5H991	Unknown	US22 family †	

US27	D[a,b]	E (Chambers et al., 1999); L (Vieira et al., 1998)	F5HDK1	Virion protein	Virion glycoprotein † GPCR family †	(Mocarski Jr, 2007)
US27	D[a,b]	E (Chambers et al., 1999); L (Vieira et al., 1998)	F5HDK1	Cell tropism/Cell type-specific replication	Deletion virus has growth defect in endothelial cells	(O'Connor and Shenk, 2011)
US27	D[a,b]	E (Chambers et al., 1999); L (Vieira et al., 1998)	F5HDK1	Viral spread	Involved in viral spread via extracellular route	(O'Connor and Shenk, 2011)
US27	D[a,b]	E (Chambers et al., 1999); L (Vieira et al., 1998)	F5HDK1	Immunomodulation	Modulation of chemo- and/or cytokine receptor	(Apweiler et al., 2004;Arnolds et al., 2013)
US27	D[a,b]	E (Chambers et al., 1999); L (Vieira et al., 1998)	F5HDK1	Modulation of host cell cycle/protein synthesis	Increase in cell proliferation and DNA replication Upregulation pro-survival transcription factors	(Lares et al., 2013)
US28	D[a,b]	E (Vieira et al., 1998;Chambers et al., 1999)	F5HF62	Modulation of host cell cycle/protein synthesis	Mediates cellular activation and migration Membrane protein † GPCR family †	(Mocarski Jr, 2007)
US28	D[a,b]	E (Vieira et al., 1998;Chambers et al., 1999)	F5HF62	Angiogenesis/Tumor formation	Promotes angiogenesis and tumor formation Membrane protein † GPCR family †	(Maussang et al., 2006;Maussang et al., 2009;Slinger et al., 2010)

US28	D[a,b]	E (Vieira et al., 1998;Chambers et al., 1999)	F5HF62	Immunomodulation	Modulation of chemo- and/or cytokine signaling<br>Membrane protein [†]<br>GPCR family [†]	(Apweiler et al., 2004;Mocarski Jr, 2007;Stropes et al., 2009)
US28	D[a,b]	E (Vieira et al., 1998;Chambers et al., 1999)	F5HF62	Gene expression/regulation	Activator of MIEP<br>Membrane protein [†]<br>GPCR family [†]	(Wen et al., 2009)
US28	D[a,b]	E (Vieira et al., 1998;Chambers et al., 1999)	F5HF62	Latency*	Transcribed in latently infected THP-1<br>Membrane protein [†]<br>GPCR family [†]	(Beisser et al., 2002;Goodrum et al., 2002)
US29	D[a,b]	E/L (Chambers et al., 1999)	F5HG95	Cell tropism/Cell type-specific replication*	May be involved in cell tropism/cell-specific replication endothelial cells<br>Putative membrane glycoprotein [†]	(Mocarski Jr, 2007)
US30	EG[a]/D[b]	E (Chambers et al., 1999)	F5HB41	Viral growth*	May be involved in temperance in fibroblasts [†]<br>Putative membrane glycoprotein [†]	(Mocarski Jr, 2007)
US31	D[a,b]	N/A	F5HAM4	Unknown	US1 family [†]	
US32	D[a,b]	L (Chambers et al., 1999)	F5HD03	Latency*	US1 family [†]	(Goodrum et al., 2002)
US33	D[a]	E (Chambers et al., 1999)	P09697 (AD169)	Unknown		
US34	D[a,b]	E (Chambers et al., 1999)	F5HEF3	Latency*	Putative secreted protein [†]	(Goodrum et al., 2002)
US34A	D[b]	N/A	Q6SVX3	Unknown	Putative membrane protein [†]	

TRS1	MGD[a]/ GD[b]	IE (Blankenship and Shenk, 2002)	Q6SVX2	(DNA) Replication	Association with UL44, essential for replication Tegument protein † US22 family †	(Marshall et al., 2009;Strang et al., 2010)
TRS1	MGD[a]/ GD[b]	IE (Blankenship and Shenk, 2002)	Q6SVX2	Gene expression/regulation	Activator IE expression Tegument protein † US22 family †	(Dunn et al., 2003;Mocarski Jr, 2007)
TRS1	MGD[a]/ GD[b]	IE (Blankenship and Shenk, 2002)	Q6SVX2	Assembly/Maturation/Egress	Role in integrity of assembly complex Capsid assembly † Tegument protein † US22 family †	(Dunn et al., 2003) (Blankenship and Shenk, 2002;Adamo et al., 2004;Mocarski Jr, 2007;Buchkovich et al., 2009)
TRS1	MGD[a]/ GD[b]	IE (Blankenship and Shenk, 2002)	Q6SVX2	Virion protein	Tegument protein † US22 family †	(Dunn et al., 2003;Mocarski Jr, 2007)
TRS1	MGD[a]/ GD[b]	IE (Blankenship and Shenk, 2002)	Q6SVX2	Immunomodulation	Inhibition autophagy Prevents activation RNase L Tegument protein † US22 family †	(Apweiler et al., 2004;Child et al., 2004;Chaumorcel et al., 2012)
TRS1	MGD[a]/ GD[b]	IE (Blankenship and Shenk, 2002)	Q6SVX2	Modulation of host cell cycle/protein synthesis	Inhibition PKR, block shut down of translation Tegument protein † US22 family †	(Apweiler et al., 2004;Cassady, 2005;Hakki et al., 2006;Mocarski Jr, 2007;Marshall et al., 2009;Child et al., 2012;Bierle et al., 2013)

IRS	D[a]	IE (Blankenship and Shenk, 2002)	Q6SW04	(DNA) Replication	Association with UL44, essential for replication Tegument protein[†] US22 family[†]	(Marshall et al., 2009;Strang et al., 2010)
IRS	D[a]	IE (Blankenship and Shenk, 2002)	Q6SW04	Gene expression/regulation	Activator IE expression Tegument protein[†] US22 family[†]	(Dunn et al., 2003;Mocarski Jr, 2007)
IRS	D[a]	IE (Blankenship and Shenk, 2002)	Q6SW04	Virion protein	Tegument protein[†] US22 family[†]	(Mocarski Jr, 2007)
IRS	D[a]	IE (Blankenship and Shenk, 2002)	Q6SW04	Modulation of host cell cycle/protein synthesis	Inhibition PKR, block shut down of translation Tegument protein[†] US22 family[†]	(Apweiler et al., 2004;Cassady, 2005;Hakki et al., 2006;Mocarski Jr, 2007;Marshall et al., 2009;Child et al., 2012;Bierle et al., 2013)
RNA2.7	N/A	E (Wu et al., 1992)	N/A	Apoptosis	Inhibitor of apoptosis	(Reeves et al., 2007)
RNA2.7	N/A	E (Wu et al., 1992)	N/A	Latency*		(Rossetto et al., 2013)
RNA1.2	N/A	N/A	N/A	Unknown		
RNA4.9	N/A	N/A	N/A	Latency*		(Rossetto et al., 2013)
RNA4.9	N/A	N/A	N/A	Gene expression/regulation		(Rossetto et al., 2013)
RNA5.0	N/A	N/A	N/A	Unknown		
IRL14	N/A	N/A	P21601 (AD169)	Unknown		

Supplementary table 1. Function annotation of the HCMV genome. **Gene**: given are the gene name based on the official gene name in the internal/terminal repeats (I/TRL), the unique long (UL) and unique short (US) regions. **Growth**: for each gene information is provided if the gene is essential (E), dispensable (D) or if deletion causes moderate growth defects (MGD), severe growth defects (SGD) or induces a growth enhancement (EG). These annotations were based on Dunn et al 2003 (a) and Yu et al 2003 (b). Yu et al. 2003 did not differentiate

between moderate or severe growth defects and thus growth defects were marked as GD. **Kinetics**: the timing of gene expression in the temporal cascade of HCMV is provided for each gene. Key: immediate-early (IE), early (E), early-late (E/L) or late (L). **Uniprot entry**: several functional annotations were based on Uniprot entries; the entry number for each gene is given. Only reviewed entries were used and unless otherwise mentioned between brackets, the entry for HCMV Merlin was used. Key: **unreviewed Uniprot entry. **Function keyword**: every gene (product) was put in a high level functional category which is the function keyword. Key: * indicates a proposed function which needs further experimental validation; $ these genes were researched in a cluster e.g. $UL118-UL119. **Process/protein/gene family**: to provide more information about the function keyword, also the process in which the gene (product) is involved is briefly described. Further, if available, information about the protein composition and/or gene family is provided. Key: † information provided by Mocarski et al. 2007, unlabeled entries are referenced in the References column; ?† annotated by Mocarski et al. 2007 with a question mark. **References**: references used for the function keyword and additional information in the process/protein/gene family column
